# Multifunctional conductive dressings for wound healing support

**DOI:** 10.1039/d6sc01545j

**Published:** 2026-05-07

**Authors:** Jingwen Yang, Lisa I. Pilkington, Jadranka Travas-Sejdic

**Affiliations:** a Centre for Innovative Materials for Health, School of Chemical Sciences, University of Auckland-Waipapa Taumata Rau 23 Symonds Street Auckland 1023 New Zealand jyan669@aucklanduni.ac.nz lisa.pilkington@auckland.ac.nz j.travas-sejdic@auckland.ac.nz; b MacDiarmid Institute for Advanced Materials and Nanotechnology Kelburn Parade Wellington 6140 New Zealand; c Te Pūnaha Matatini Auckland 1142 New Zealand

## Abstract

Chronic and non-healing wounds, characterised by persistent inflammation, recurrent infections and impaired angiogenesis, remain a significant clinical and socioeconomic burden. Conductive polymers (CPs) have emerged as promising materials for actively accelerating wound repair owing to their unique combination of electroactivity, redox activity and tunable physiochemistry. This review highlights recent advances in the use of CPs, such as polypyrrole (PPy), polyaniline (PANI) and poly(3,4-ethylenedioxythiophene) (PEDOT), for chronic wound management, by means of electrical stimulation therapy, electrochemically controlled therapeutic delivery and wound monitoring. CP-based wound dressings have demonstrated significant potential to promote tissue regeneration, modulate inflammation, improve infection control and reduce pathological scarring in chronic wounds. In this review, chemical and electrochemical synthesis of CPs and processing methods for CP-based wound dressings are outlined, before focusing on CP-based, on-demand drug delivery systems and electrical stimulation for wound healing. Emphasis is then placed on multifunctional systems for enhanced therapeutic efficacy. We also briefly outline the recent progress toward the transition from externally programmed electrical stimulation to self-powered integrated systems. Finally, we discuss current challenges in developing CP-based wound dressings, such as long-term stability, multifunctionality and clinical translation, and outline future directions toward intelligent, personalised wound-care systems based on CP-based wound dressings.

## Abstract 2

Advanced multifunctional conductive polymer-based wound dressings integrate biosensing, on-demand drug release and electrical stimulation to enable real-time monitoring of the wound status and adaptive, closed-loop wound management.

## Graphical abstract

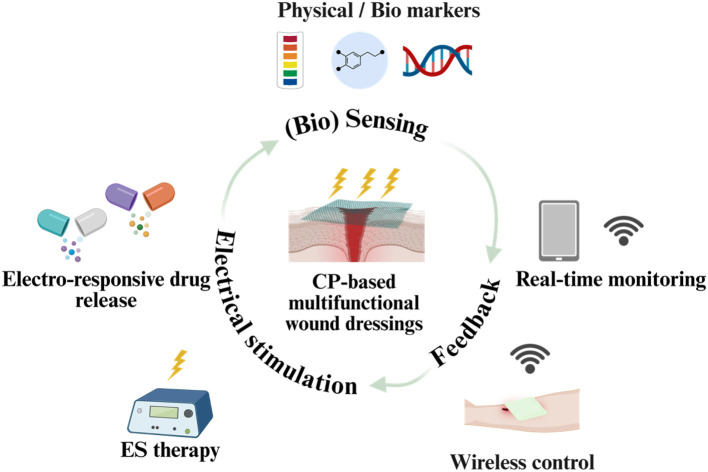

## 1. Introduction

**Fig. 1:**
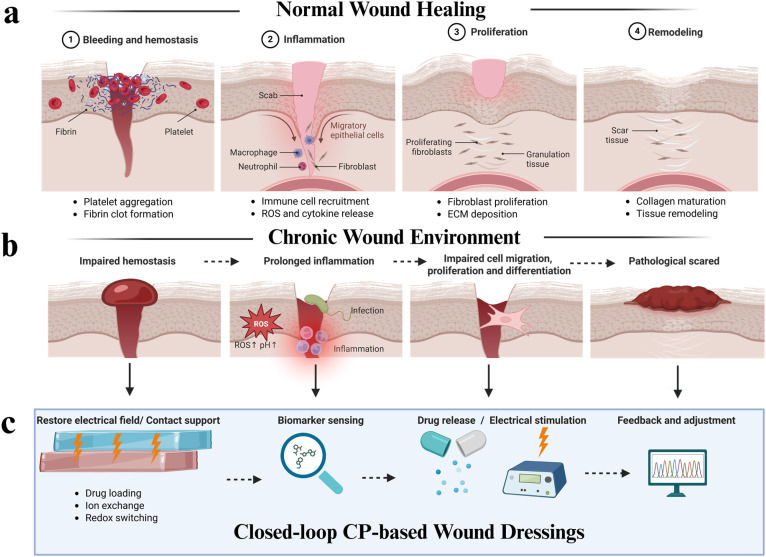
(a) Timeline of the wound-healing cascade (haemostasis, inflammation, proliferation and remodelling), (b) characteristics of chronic wound environment and (c) stage-specific chemical interventions enabled by CPs, including biomarker sensing, electrochemically controlled drug release and electrical stimulation, for closed-loop regulation of chronic wound environments. Created in BioRender. Yang, J. (https://BioRender.com/ijqo26j) is licensed under CC BY 4.0.

Wound physiology encompasses a complex, sequential cascade of cellular and molecular events to restore tissue integrity, following injury. This dynamic process involves four continuous and overlapping phases (Fig. 1a): haemostasis, inflammation, proliferation and remodelling.^1^ Haemostasis is rapidly initiated post-injury and is characterised by platelet activation and fibrin clot formation to halt bleeding and provide a provisional extracellular matrix. Subsequently, inflammatory cells infiltrate the wound site, releasing cytokines and growth factors that regulate tissue defence and initiate repair mechanisms. The proliferative phase follows, marked by fibroblast migration, extracellular matrix deposition, angiogenesis and re-epithelialisation, collectively facilitating the formation of granulation tissue. Finally, during the remodelling phase, collagen fibers are reorganised and cross-linked, resulting in scar maturation and restoration of functional tissue architecture. A variety of biological agents' activities help with these different stages, including cytokines (anti-inflammatory and pro-inflammatory) and growth factors. However, wound repair does not always progress in a timely or orderly manner.

Acute wounds, such as incisions or excisions, typically follow the normal healing trajectory, whereas chronic wounds exhibit dysregulated repair.^2^ Chronic wounds are characterised by a persistently dysregulated microenvironment, including prolonged inflammation, elevated levels of reactive oxygen species (ROS), bacterial infection, impaired angiogenesis and disrupted cellular signalling. Such pathological conditions are commonly associated with diabetes,^3^ burns, skin cancer^4^ and infection,^5^ as well as factors like aging or inappropriate clinical management, that often lead to impaired healing (Fig. 1b). Chronic wounds that remain unhealed for months or even years are extremely burdensome on the individual and their families, affecting quality of life and the healthcare system.

Traditional wound dressings primarily function to shield the wound from infection and preserve a moist environment that supports re-epithelialization. However, these dressings only provide coverage of the wound and often cause disruption and pain upon removal.^6^ In contrast, modern multifunctional biomaterial-based wound dressings designed to maintain a moist environment, manage exudate, protect against pathogens and provide antibacterial and antioxidant activity, self-healing capacity, adhesiveness and appropriate mechanical properties, have recently emerged and demonstrated clear advantages in complex wound-healing applications.^7–9^

Conductive polymers (CPs) represent a promising class of materials for smart wound care applications. CPs, such as polypyrrole (PPy), polyaniline (PANI) and poly(3,4-ethylenedioxythiophene) (PEDOT), possess a π-conjugated backbone that enable reversible doping/dedoping, efficient mixed electronic–ionic transport and redox-responsive induced changes (*e.g.* volume, charge, hydrophilicity).^10–12^ These properties enable CPs to function not only as passive scaffolds but also as active bioelectronic interfaces. As depicted in Fig. 1, CP-based wound dressings enable stage-specific chemical interventions that directly target the pathological features of chronic wounds. Three key therapeutic functions can be achieved in CP-based wound dressing (Fig. 1c): (i) transduction of biological signals into electrical outputs for real-time monitoring; (ii) electrochemical delivery of therapeutics with programmable spatiotemporal control; and (iii) mediation of electrical and electrochemical stimulation (ES) at tissue-relevant current densities. Importantly, the integration of these functionalities within a single platform establishes the foundation for closed-loop wound management systems, in which sensing, actuation and therapeutic delivery are dynamically coupled.

CPs composite with other (bio)polymers could improve surface conformability and biocompatibility of CP-based wound dressings while preserving electroactivity.^13,14^ CP surfaces can also be molecularly modified and functionalised, *e.g.* utilising extracellular matrix (ECM)-derived dopants,^15^ peptide grafts,^16^ or antifouling brushes,^17^ to modulate protein adsorption or cell–material interactions, reduce biofouling and enhance integration with skin tissue. Various fabrication strategies enable CP-based dressings to be produced into forms tailored to wound geometry, such as films,^18^ hydrogels,^19^ fiber mats,^20^ microneedle (MN) arrays^21^ and three-dimensional (3D) composites.^22^

CPs have shown great potential to afford controlled and on-demand drug release. Due to their unique electrical and physicochemical properties, and the fact that they are easily synthesised, CP properties can be precisely tuned by adjusting synthesis parameters, and they can be integrated with other materials to form composite systems with tailored functionalities.^23^ CPs can have high surface area, good charge storage capacity, and high conductivity which make them suitable for the controlled release of drugs.^24,25^ In parallel, CPs serve as effective interfaces for ES therapy, where their soft, conductive and conformable nature allows efficient coupling of electrical cues with the wound surface. By mimicking or amplifying endogenous electrical fields (EFs) at the wound site, ES delivered through CP-based dressings has been shown to promote key wound-healing processes, including cell migration, proliferation, angiogenesis and modulation of inflammatory responses.^26^ Notably, a synergistic effect on wound healing can be potentially achieved through the integration of drug delivery and ES therapies. For instance, PEDOT-based hydrogel has been shown to enhance the release of a therapeutic agent (insulin) across pig skin under the application of ES.^27^ In addition, CPs enable continuous, non-invasive monitoring of wound biomarkers.^28^ Therefore, CPs show a promise as multi-functional systems that can provide ES therapy, electro-responsive drug delivery and biosensing in one system, to address the diverse challenges of diabetic, infected, delayed-healing and scar-prone wounds. Recent advances in CP-based wound dressings have also enabled integration with emerging self-powered configurations to reduce reliance on external power sources while preserving soft, conformable and wearable architectures.

This review provides a comprehensive overview of recent advances in CP-based multifunctional wound dressings for chronic wound management. Focusing on representative CPs, such as PPy, PANI and PEDOT, we examine how their unique electrochemical properties enable electrically mediated therapeutic delivery, ES to promote tissue regeneration and real-time wound monitoring through biosensing. We first outline the electrochemical synthesis and fabrication strategies underpinning CP electroactivity and processability into wound-dressing formats. We then discuss CP-based, on-demand drug delivery systems, electrical stimulation-enhanced wound healing and emerging multifunctional platforms that synergistically integrate stimulation, controlled release and sensing to enhance therapeutic efficacy. Finally, we address key challenges, including long-term stability, multifunctional integration and clinical translation, and highlight future directions toward intelligent, personalised and closed-loop wound-care systems enabled by CP-based wound dressings.

## 2. Conductive polymers for wound dressings

### 2.1 Conductive polymers' chemistry and implications for wound-healing applications

**Fig. 2:**
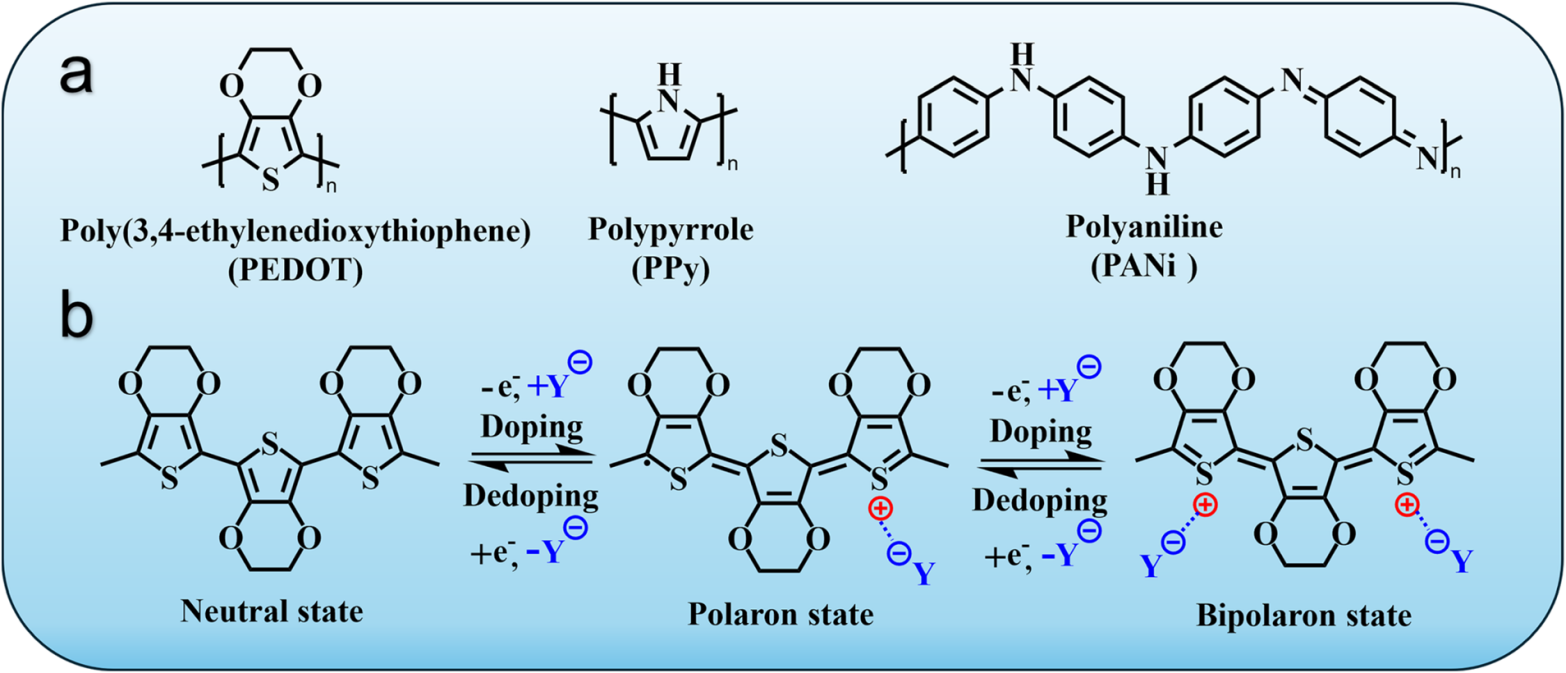
Fundamental chemistry and redox mechanisms of conductive polymers. (a) Chemical structures of commonly used conductive polymers, including poly(3,4-ethylenedioxythiophene) (PEDOT), polypyrrole (PPy) and polyaniline (PANI). (b) Doping mechanism of conductive polymers exemplified by PEDOT. The π-conjugated polymer backbone enables charge delocalisation along the chain. Oxidation (p-doping) is balanced by counter-ion ingress (Y^−^) forming polarons and bipolarons, whereas reduction expels ions and restores neutrality. Redox-driven ion exchange and polymer swelling support electrically triggered drug release and mixed ionic – electronic conduction, forming the chemical basis of multifunctional wound-healing systems.

Conductive polymers (CPs) are conjugated organic macromolecules in which π-electron delocalization along the polymer backbone enables charge transport through overlapping orbitals. The key feature distinguishing CPs from conventional polymers is the presence of alternating single and double bonds, which support the formation of mobile charge carriers upon oxidation or reduction.^10,29^ Typical CPs, such as polypyrrole (PPy), polyaniline (PANI) and poly(3,4-ethylenedioxythiophene) (PEDOT), are synthesised *via* chemical or electrochemical oxidation polymerisation of their heteroaromatic monomers (Fig. 2). Their electrical conductivity, which can span orders of magnitude (typically 10^−5^–10^3^ S cm^−1^), depends on the extent of conjugation, oxidation state and counter-ion incorporation.^30,31^

#### 2.1.1 Doping chemistry of CPs

The electrical activity of CPs arises from a doping–dedoping process, in which the conjugated backbone is oxidised (p-doping) or reduced (n-doping) and charge-compensated by the ingress or egress of counter-ions from the surrounding medium.^32,33^ This process generates quasi-particles such as polarons and bipolarons, whose delocalisation governs electronic conductivity. The redox reversibility of this mechanism allows dynamic modulation of charge density, ionic flux and polymer conformation under electrical bias, features directly relevant to electrically triggered drug release and electromechanical actuation in wound dressings.^34,35^

The type and size of dopant anion significantly influence conductivity, morphology and biocompatibility. Small dopants (*e.g.*, Cl^−^, PF_6_^−^) enable high conductivity but limited stability in aqueous media, whereas bulky or polymeric dopants (*e.g.*, *p*-toluenesulfonate, heparin, poly(styrenesulfonate) (PSS)) enhance aqueous stability, swelling control and biological tolerance.^36,37^ In PEDOT:PSS, for instance, the sulfonate groups of PSS acts as fixed anionic dopants that stabilise PEDOT^+^ chains while imparting hydrophilicity and processability in aqueous dispersion – key features for biomedical and wound-care applications.^38^

When a potential is applied, the redox cycling of CPs drives the insertion and expulsion of ions and associated solvent molecules to maintain charge neutrality. This coupling between electronic and ionic transport gives CPs their mixed conduction character.^39,40^ In aqueous or physiological media, cations (*e.g.*, Na^+^, K^+^, H^+^) and anions (*e.g.*, Cl^−^, SO_4_^2−^) can move within the polymer matrix through micro- or nano-porous domains formed during polymerisation. The resulting volumetric swelling or contraction alters polymer permeability and mechanical properties, which can be harnessed to modulate the release of therapeutics incorporated as mobile dopants or encapsulated within the matrix.^41,42^ This redox-driven ion exchange underpins the operation of electroresponsive wound dressings, where a controlled potential can trigger the release of bioactive species such as antibiotics, anti-inflammatory agents or growth factors.^43–46^

The interplay between polymer backbone structure, dopant chemistry and morphological organisation governs the electrochemical performance and biocompatibility of CPs. Extended conjugation and planarity promote charge delocalisation, while heteroatom substitution (*e.g.*, nitrogen, oxygen, sulphur) modulates ion affinity and redox potential.^47,48^ Incorporating hydrophilic side chains, grafting biopolymers or forming block copolymers can improve aqueous dispersibility, mechanical compliance and interaction with biological tissues.^49–51^

From a design standpoint, the challenge lies in balancing electrical performance, chemical stability and biological safety. Overoxidation of CPs, especially PPy and PANI, can disrupt conjugation and degrade conductivity, whereas excessive doping may induce cytotoxicity.^52,53^ Recent strategies address these issues by using biocompatible dopants, cross-linked or composite architectures and *in situ* polymerisation on biopolymeric scaffolds, which collectively stabilise the redox state and mechanical integrity under physiological conditions.^54–56^

#### 2.1.2 Comparative chemical and electrochemical properties of CPs

Despite the shared doping mechanisms, PPy, PANI and PEDOT show distinct constraints on oxidation tolerance, ion transport and environmental stability, which ultimately dictate their suitability for different bioelectronic functions. To compare these widely used CPs, a summary of their key electrochemical, physicochemical and biological properties is provided in Table 1. PPy exhibits excellent chemical stability in air and water, making it resistant to environmental factors, and able to maintain reasonably high conductivity under physiological conditions, with values 10–50 S cm^−1^.^57^ PPy can be synthesized easily and in large quantities at room temperature in various solvents, including water. Its high surface area and tunable porosity facilitate the incorporation of bioactive molecules, making it a suitable platform for drug delivery and other biomedical applications.^58^ However, PPy also presents several intrinsic limitations. It is non-thermoplastic, mechanically rigid and brittle, and typically insoluble in common solvents, which restricts its processability.^59^ The presence of ‘disorder’ or defective regions within the PPy can limit its conductivity; such defects may arise from the overoxidation (typically above ∼0.65 V) in the presence of oxygen and water, gradually degrading its electrical properties.^60^ PANI shows electrical conductivity ranging from 0.1–5 S cm^−1^, which depends on its oxidation state, polymer crystallinity and conjugation length.^61^ However, the conductivity of PANI is strongly pH-dependent, as deprotonation under neutral or alkaline conditions leads to a significant loss of electrical activity, which limits its stability and performance in physiological environments.^62^ Through the use of strong polyacid dopants and composite architecture, PANI's electroactivity can be maintained in physiological environments.^63^ PANI also has low processability due to the rigidity of its polymer backbone, which makes it insoluble and non-meltable.^64^ PEDOT, a derivative of polythiophene, exhibits superior electrochemical stability, high electrical conductivity, and excellent thermal stability. These properties render PEDOT particularly well-suited for applications requiring long-term durability, such as in implantable bioelectronic devices. Furthermore, PEDOT supports coupled electronic and ionic transport, enabling efficient mixed conduction.^35^ This characteristic facilitates effective electrical signal transduction and interface compatibility within biological systems for stimulation. However, PEDOT is limited by its low solubility and poor processability. PEDOT's processability is notably enhanced when combined with additives/dopants like polystyrene sulfonate (PSS), which render it water-soluble. In PEDOT: PSS, the structure forms a micelle with a hydrophobic PEDOT-rich core and a hydrophilic PSS-rich shell. This structure helps to stabilize the PEDOT-enriched particles in aqueous solvents.^35^

**Table 1:** Comparative chemical and electrochemical properties of representative conductive polymers (PPy, PANI, PEDOT) and their implications for wound-healing applications

CPs	Doping level	Maximum potential for polymerisation (V)	Conductivity (S cm^−1^)	Capacitance (F g^−1^)	Processability	Chemical stability in aqueous environments/limitations	Wound healing applications	Ref.
PPy	0.33	0.8	10–50	530–620	Poor (insoluble, and infusible)	Prone to overoxidation, loss of conductivity and structural degradation	Drug delivery (high dopant loading capacity)	57
PANI	0.5	0.7; pH-dependent	0.1–5	240–750	Poor (rigid backbone, limited solubility)	pH-dependent, loss of conductivity at neutral/alkaline conditions	pH sensing	61
PEDOT	0.33	1.2	300–500	92–210	Good when formulated as PEDOT:PSS (aqueous dispersibility)	Stable backbone and low oxidation potential, enabling sustained conductivity in physiological media, low drug loading capability	Electrical stimulation & sensing	65 and 66

Collectively, PEDOT exhibits the most favourable balance of electrochemical stability, low oxidation potential and mixed ionic–electronic conduction, making it particularly suitable for electrical stimulation and bioelectronic interfaces. In contrast, PPy offers high drug-loading capacity and facile synthesis, rendering it advantageous for electrochemically controlled drug delivery systems. PANI, while exhibiting tunable redox behaviour, is limited by its pH-dependent stability and is therefore more suitable for sensing applications under controlled conditions. These intrinsic differences highlight the importance of rational CP selection based on the specific functional requirements of wound-healing applications.

The chemical versatility and redox-active nature of CPs directly translate to functional benefits in wound-care systems. Their tunable conductivity allows modulation of endogenous electric fields to promote cell migration and angiogenesis, while redox-triggered ion flux enables controlled therapeutic release^67,68^ and real-time electrochemical sensing.^69^ The capacity to couple these effects within a single polymeric framework makes CPs unique among bioelectronic materials and provides the chemical foundation for the multifunctional wound-healing platforms discussed in subsequent sections.

### 2.2 Synthesis of CPs, composites and fabrication strategies for wound dressings

**Fig. 3:**
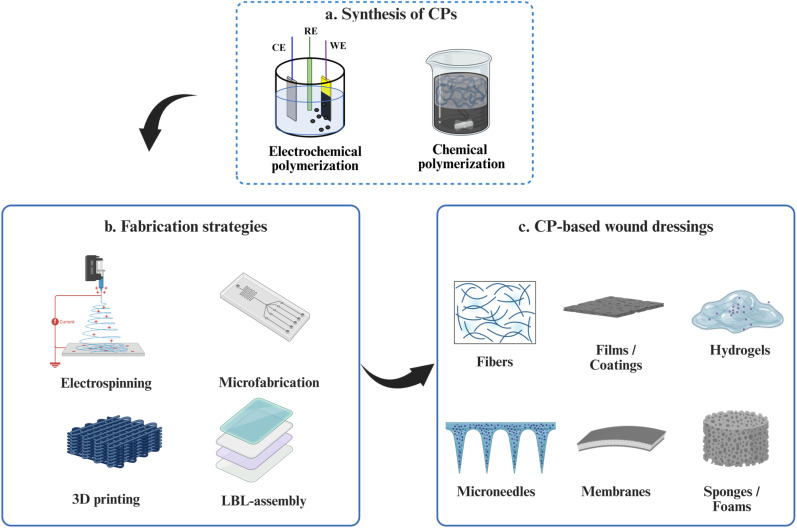
Design framework for CP-based wound dressings. (a) Synthesis of CPs, (b) fabrication strategies and (c) presentation of CPs in wound dressings. Created in BioRender. Yang, J. (https://BioRender.com/6a9z9vv) is licensed under CC BY 4.0.

The synthesis and fabrication of CPs play a decisive role in controlling their microstructure, conductivity and biocompatibility, parameters that directly dictate performance in wound-healing platforms.^70,71^ Two complementary levels of control are generally distinguished, as shown in Fig. 3: (a) molecular-level synthesis, defining polymer backbone, dopant and oxidation state; and (b) CPs fabrication, dictating film morphology, porosity and interface with biological matrices. A rational combination of these approaches enables CPs to be engineered into mechanically compliant, electrochemically stable and biologically interactive dressings with different architectures (c).

#### 2.2.1 Chemical and electrochemical polymerization, and interfacial, mechanical and architectural design considerations

Most CPs used in biomedical systems are produced either by chemical oxidative polymerisation or electrochemical polymerisation.^72^

Chemical oxidative routes employ oxidants, such as FeCl_3_, ammonium persulfate or H_2_O_2_, to convert monomers (pyrrole, aniline, thiophene, EDOT) into conjugated chains. Reaction parameters – monomer/oxidant ratio, solvent polarity, temperature and dopant species – influence molecular weight and doping level. Incorporation of biocompatible dopants (*e.g.*, *p*-toluenesulfonate, heparin, dextran sulfate) yields water-dispersible and cytocompatible CPs suitable for hydrogel or composite formulations.^73^ However, chemical polymerisation offers limited control over the polymerisation process and redox state of the synthesised polymer, which makes it difficult to achieve a uniform morphology, specific molecular weights and desired doping levels.^74^

Electrochemical polymerisations, typically carried out in aqueous or mixed electrolytes under potentiodynamic, potentiostatic, or galvanostatic control, afford thin films with precise thickness and oxidation state.^75,76^ This approach provides superior control over film thickness and uniformly deposited CPs on conductive substrates,^13^ yielding conformal coatings on electrodes, microneedles or fibrous scaffolds without additional binders. Electrochemical synthesis also enables *in situ* doping with therapeutic anions (*e.g.*, salicylate, dexamethasone, gentamicin), imparting immediate drug-loading functionality.^77,78^ Such films can subsequently release the incorporated agents through potential-controlled ion exchange, integrating fabrication and functionalisation in a single step.

To improve interfacial adhesion and biocompatibility, CPs are frequently polymerised *in situ* within or upon biopolymeric substrates such as chitosan, gelatin, collagen, silk fibroin or bacterial cellulose.^79,80^ This approach enables interpenetration of polymer networks, producing mechanically robust composites with enhanced ionic conductivity and moisture retention. Surface-initiated oxidative polymerisation of pyrrole or EDOT onto carboxylated or amine-functionalised polysaccharide matrices generates continuous CP coatings while preserving the substrate's micro-porous architecture essential for cell infiltration and gas exchange.^81,82^ Chemical coupling agents (*e.g.*, 1-ethyl-3-(3-dimethylaminopropyl) carbodiimide) (EDC)/*N*-hydroxysuccinimide (NHS) or plasma activation are often used to anchor monomers to the substrate, preventing delamination during redox cycling.^83,84^ These strategies also reduce overoxidation by spatially confining charge propagation and stabilising dopant distribution.^85^

CP composites offer a powerful means to couple electrical, mechanical and biological functionalities. Combining CPs with inorganic or organic fillers offers a versatile route to tailor conductivity, mechanical strength and biological response.^86^ Nanostructured fillers, such as graphene, carbon nanotubes, silver nanowires (NWs) or bioactive ceramics, can provide efficient pathways for charge transport and reinforce tensile properties,^87–89^ while metal nanoparticles (NPs) such as Ag, Au and Cu impart potent antibacterial effects, although their cytotoxicity and rigidity necessitate careful optimisation.^90^ Integration of CPs with biodegradable polymers such as polycaprolactone (PCL), polylactide (PLA) or polyurethane yields elastic, breathable and degradable films suitable for wound coverage.^91^

Post-synthetic treatments further refine CP performance. Solvent treatments with dimethyl sulfoxide (DMSO), ethylene glycol or surfactants reorganise the morphology of CPs, increasing phase separation and carrier mobility.^92^ For instance, addition of DMSO to PEDOT:PSS improves electrical conductivity by inducing conformational changes and phase separation between PEDOT and PSS.^93^ Thermal annealing or mild acid/base washing can remove residual oxidants and tune the oxidation level of PEDOT:PSS, leading to improved electrical conductivity and stability.^94^ In biological contexts, these steps also lower cytotoxicity and minimise leaching of low-molecular-weight dopants.^95^ Redox-exchange doping, wherein dopant ions are replaced post-synthesis with biologically relevant species (*e.g.*, phosphate, ascorbate, growth-factor polyanions), is another simple approach to impart biochemical functionality to CPs.

Effective CP-based wound dressings must maintain intimate yet non-damaging contact with the wound bed while resisting biofouling and mechanical failure. These interfacial properties are commonly engineered by integrating proteins, polysaccharides or biocompatible synthetic polymers as dopants, composite matrices or surface-modification (such as coating, chemical grafting and functionalising) within CPs.^79,96^ Protein-based materials (fibrin, albumin, gelatin) exhibit strong tissue adhesion and mimic native extracellular matrix (ECM).^97–99^ Polysaccharides, such as polydopamine (PDA), hyaluronic acid (HA), dextran, chitin, chitosan (CS) and chondroitin sulfate, offer biocompatibility, chemical versatility, and in some cases intrinsic antimicrobial activity.^79,80,100,101^ Synthetic hydrogels (*e.g.*, poly(ethylene glycol) (PEG), poly(vinyl alcohol) (PVA)) are often used as CP composite matrices or interpenetrating networks to provide controlled degradation, high water content and mechanical compliance compatible with soft tissues.^102^ Notably, the incorporation of large biomolecular dopants can compromise coating cohesion and interfacial adhesion, increasing the risk of delamination under mechanical or electrochemical stress.^103^ Therefore, careful molecular and interfacial design is required to balance bifunctionality with long-term mechanical integrity.

Antifouling performance, while preserving electroactivity, can be achieved through molecular engineering of CP backbones, including polyglycerol-, polyethylene glycol-, or zwitterion-functionalised PEDOT derivatives.^104–108^ Thiolated hyaluronic acid (THA) has recently emerged as a promising polymer for designing mucoadhesive and antifouling interfaces enabled by its integration with CPs *via* dopant incorporation or covalent grafting.^109–111^ In particular, THA could be potentially covalently coupled with thiolated CPs through reversible disulfide bond formation, allowing electrochemical modulation of interfacial properties.^112–114^ Notably, the redox state of CPs can influence microbial adhesion; *e.g.* it was demonstrated that reduced PEDOT surface exhibit diminished interactions with respiring bacteria.^115^

Mechanical properties are tuned through polymer composition, dopant selection, crosslinking density and ‘architecture’ (see below). Blending CPs with soft polymers or elastomers, including polyurethane (PU), polyethylene oxide (PEO), poly(methacrylic acid) (PMAA), poly(acrylic acid) (PAA) and poly(dimethylsiloxane) (PDMS), could be used in fabricating highly stretchable, conductive films.^116,117^ The incorporation of flexible dopants or plasticizers (*e.g.*, sulfonated biomolecules: PSS, d-mannitol or ionic liquids) into intrinsically rigid CP effectively reduces brittleness while preserving electrical conductivity.^54^ CP-hydrogel hybrid or composite systems, incorporating gelatin, alginate, CS or cellulose, further enhance moisture retention and fatigue resistance, while maintaining sufficient charge transport for ES or sensing.^118–121^ Crosslinking strategies in CP hydrogels must balance mechanical robustness with electrical continuity, where permanent covalent bonds usually result in a tough matrix, while physical crosslinking methods, including ionic interactions and hydrogen bonding, can impart self-healing and stimuli-responsive features.^122^ Modulation of crosslinking density further enables the adjustment of elastic modulus to better match native skin or wound tissue, thereby minimizing mechanical mismatch and irritation.^123^

The architecture of CP-based wound dressing also plays a pivotal role. Commonly, film and fibers have good oxygen permeability, resistance toward water and tough mechanical properties. Microneedle arrays enable mechanical robustness combined with minimally invasive penetration.^124^ Sponges, foams and hydrogels, owning to their 3D network and porous structure, could absorb large amount of exudate, maintain moist environment and act as carriers for bioactive substances and cells.^125,126^

#### 2.2.2 Fabrication strategies for CP-based wound dressings

CPs are inherently challenging to process due to their limited solubility and processability, hydrophobicity and inability to degrade.^127^ To overcome these limitations, a widely adopted strategy involves applying conductive CP coatings onto non-conductive, flexible and structurally engineered substrates, which provides an effective alternative route for fabricating functional wound dressings.^128,129^ Advanced processing techniques, including electrospinning, additive manufacturing (AM), microfabrication and layer-by-layer (LbL) assembly, enable the fabrication of customised hierarchical structures and have the potential to accommodate high loads of therapeutics.^130^

Electrospinning produces fiber mats with micro- to nano-scale diameters that mimic the structural and mechanical features of the native ECM, thereby providing a microenvironment that supports cell adhesion, migration and tissue regeneration.^131,132^ Electrospun PEDOT- or PANI-based composites (*e.g.*, PEDOT:PSS/chitosan^20^ or PANI/gelatin^133^), retain electrical conductivity while allowing for tunable fiber diameter, alignment and porosity to modify the electrochemical activity, drug-loading capacity and release profiles.^134^ Coaxial electrospinning and triaxial electrospinning strategies further enable compartmentalised architectures for sequential or stimulus-responsive therapeutic release.^135^

3D printing and AM technologies also provide options for programmable porosity, mechanical gradients and patient-specific geometries.^136^ These techniques facilitate fabrication of personalised wound dressings tailored to wound size, depth and healing stage.^137^ AM technologies, including inkjet, extrusion, electrohydrodynamic and light-based printing, further enable CPs to be combined with other polymers and/or conducting fillers, such as carbon nanotubes, graphene and silver NWs, to produce complex materials and designs^89^ including hydrogels, elastomers and drug reservoirs, within a single construct.^138^ Such capabilities are particularly advantageous for embedding stimulation electrodes, biosensors and drug reservoirs directly into the dressing matrix.^139^ Notably, recent advances in 3D bioprinting have enabled CP-based wound dressings to mimic dermal matrices while incorporating living cells.^139^ CP bioinks are typically formulated by blending CPs with viable cells, growth factors, cytokines and other biocompatible polymers, followed by mild solidification or crosslinking processes that preserve cell viability while stabilizing the printed architecture.^139^ PEDOT:PSS with GelMA^140^ or sodium carboxymethyl cellulose (CMC)/ALG^141^ has been used for bioprinting and showed low impedance and nearly 100% bioprinted fibroblast cells viability. When combined with ES, the bioinks significantly enhanced the elongation and proliferation of human skin fibroblasts.^141^

Patterned CP architectures fabricated *via* printing and microfabrication techniques offer additional opportunities for spatially controlled stimulation and sensing. Solution-processable CP formulations, most commonly based on PEDOT:PSS^142,143^ or PANI,^144,145^ can be deposited onto flexible substrates such as polyurethane films, electrospun polycaprolactone (PCL) mats, bacterial cellulose or hydrogel matrices using scalable methods including screen printing, inkjet printing, dip coating and spray coating. These approaches enable highly effective, low-cost and scalable fabrication of mechanically compliant and disposable wound dressings while allow the direct embedding of conductive circuits.^146^ Through rational optimisation of CP formulations, these approaches further allow patterned incorporation of drug-loaded CP domains,^147^ enabling localised therapeutic delivery and enhanced wound-healing efficacy.

Advanced microfabrication techniques (*e.g.*, soft lithography, laser cutting and photolithography) allow the fabrication of the surfaces with well-defined microstructures like microfluidic channels, sensor arrays and microneedle architectures.^148,149^ CP-based sensor arrays integrated with microfluidic systems enable efficient collection and analysis of wound exudate and simultaneous and multiplexed detection of biomarkers.^150^ For example, multiplexed infection monitoring was demonstrated using a multimodal sensor system based on laser-induced graphene (LIG) in which PPy was electrochemically deposited on the porous LIG electrode, thereby enhancing electrochemical drug loading and sensing performance of wound-relevant biomarkers.^151,152^ In addition, micro- and nanostructured CPs, such as PPy NWs,^153^ PEDOT nanotubes^154^ and PANI microneedles,^155^ have been shown to increase electrochemically active surface area, enhance redox efficiency, and facilitate localised delivery of therapeutic agents.

Layer-by-layer (LbL) assembly provides a complementary route for constructing multifunctional CP-based wound dressings. Sequential deposition driven by electrostatic, hydrogen-bonding, hydrophobic or covalent interactions enables precise control over CP film composition and thickness.^156^ Each layer can be independently engineered to serve a specific function, such as drug loading and release, energy harvest, ion transport, exudate absorption, biosensing or electrical stimulation. This modularity is particularly attractive for designing multifunctional wound dressings.

In summary, by applying chemical/electrochemical polymerisation, composite formation and architectural organization, CP-based systems can be rationally engineered to transition from passive wound coverings to active, adaptive therapeutic platforms. The following sections discuss the trigger mechanisms and functional capabilities of CP-based wound dressings, with particular emphasis on electrical stimulation, electrochemically controlled drug delivery and wound sensing.

## 3. CP-based dressings for electrical stimulation

Endogenous bioelectric signals play a critical regulatory role during skin repair. Intact human skin maintains a transepithelial potential generated by asymmetric ion transport across the epidermis, effectively functioning as an endogenous ‘battery’.^157^ Disruption of the epidermal barrier upon injury results in the formation of lateral electric fields (EFs) at the wound margins, which serve as directional cues for keratinocyte, fibroblast and endothelial cell migration, thereby coordinating re-epithelialization, angiogenesis and tissue organization through electrotaxis.^158^ In chronic and non-healing wounds, including diabetic, ischemic and infected ulcers, these endogenous EFs are often attenuated or spatially disordered due to prolonged inflammation, impaired ion transport and tissue necrosis. Exogenous ES is therefore applied to restore, amplify or spatially regulate bioelectric cues to re-establish a pro-healing microenvironment.^159^

Conventional metal-based electrodes, including metals, metal oxides, alloys and their composite nanostructures, offer rapid electron transfer which are advantageous for electrical signal delivery. However, they often suffer from corrosion, during electrochemical processes, which can significantly deteriorate ES performance.^160^ Moreover, their mechanical rigidity results in a pronounced mismatch with soft biological tissues, hindering conformal contact with irregular wound surfaces and consequently reducing stimulation efficiency.^161^ Carbon-based materials, such as carbon nanotubes (CNTs), graphene derivatives (*e.g.*, reduced graphene oxide, rGO), and MXenes (*e.g.*, Ti_3_C_2_T_*x*_), have emerged as alternative conductive materials for wound healing platforms due to their stable electrochemical properties, wide electrochemical windows and fast electron transfer kinetics.^162–164^ Nevertheless, their electrochemical behaviour and structural characteristics introduce additional challenges for wound applications. CNT- and graphene-based electrodes typically require relatively high applied potentials (often exceeding ±1.0 V) to achieve sufficient charge injection, which increases the risk of parasitic faradaic reactions, including water electrolysis and reactive oxygen species (ROS) generation.^165,166^ MXenes, despite their exceptionally high conductivity (10^2^ to 10^4^ S cm^−1^),^167^ are susceptible to oxidative degradation in aqueous and oxygenated environments, particularly at anodic potentials above +0.2 to +0.4 V (*vs.* Ag/AgCl), thereby limiting their long-term electrochemical stability under physiological conditions.^168^ Furthermore, issues on stiffness, flexibility, conductivity retention and performance under high-frequency cyclic strains, pose additional challenges.^169^ In contrast, CPs provide a more suitable materials platform for bioelectronic wound applications by addressing both electrochemical and mechanical constraints of other conducting materials. Importantly, CPs operate within physiologically compatible electrochemical windows (typically −0.6 to +0.5 V *vs.* Ag/AgCl),^170^ enabling effective charge delivery at substantially lower voltages. This advantage is further reinforced by intrinsic mixed ionic–electronic conduction and high volumetric charge storage capacity of some of CPs, which facilitate efficient, predominantly capacitive charge injection with high charge injection capacity (CIC). CPs can be integrated into soft, flexible substrates and engineered as continuous films or patterned architectures to tailor the electric field distribution and conformal contact. The electrode patterns can be designed to align parallel to the wound edges to generate a uniform electric field across the wound.^171,172^ Moreover, voltage applied on CPs can be systematically varied over a wide potential range and CPs can store significant charge and inject low currents (nA to pA) at short timescales (ms).^74^ This is important since these features decrease the electrode polarization and generated heat during stimulation, thereby providing safer ES.

Safe and effective ES requires the controlled activation of excitable cells by mimicking the wound's endogenous electric field (10–60 mV across the wound, peaking at 100–200 mV mm^−1^ near the edge).^173^ This necessitates the use of lower stimulation voltages to achieve optimal electrical activation. Yu *et al.*^174^ demonstrated that PEDOT:PSS/PVA hydrogel coating exhibited an electrical conductivity of over 9 S m^−1^, a low impedance (<30 Ω) in PBS buffer at the physiologically-relevant frequencies (10^2^–10^5^ Hz), as well as high CIC up to 4.4 mC cm^−2^, under ES (−0.5 to 0.5 V *vs.* Ag/AgCl). Notably, compared with metal electrodes, CP electrodes exhibit significantly lower interfacial impedance due to their mixed ionic–electronic conduction, enabling more efficient charge transfer and improved bioelectrical coupling with soft tissues. For example, structured CP architectures, such as 3D PEDOT pillars show substantially enhanced charge storage (up to 127 ± 5.6 mC cm^−2^) compared with planar Au electrodes (9.5 ± 0.3 mC cm^−2^), which is attributed to their high surface area and reduced impedance.^175^

Despite these advantages, the electrochemical safety window of CP-based systems in physiological environments must be carefully considered. In aqueous biological media, the applied potential should remain within a range that avoids parasitic faradaic reactions, particularly water electrolysis and the unintended oxidation of endogenous biomolecules. Water electrolysis typically occurs at potentials beyond approximately −0.6 V to −0.9 V (reduction) and +0.6 V to +0.8 V (oxidation) *vs.* Ag/AgCl, leading to hydrogen or oxygen evolution and associated local pH changes.^176^ In addition, electroactive species naturally present in wound exudate, such as ascorbic acid and uric acid, can undergo oxidation at relatively low potentials (0.1–0.5 V), potentially generating reactive intermediates and disrupting redox homeostasis.^177^ These processes may result in local acidification or alkalisation, generation of ROS and oxidative damage to cells and extracellular matrix components, ultimately impairing wound healing. Accordingly, a practical safe electrochemical window for CP operation is generally considered to lie within approximately −0.6 to +0.5 V *vs.* Ag/AgCl, although this range depends on electrolyte composition, oxygen content and device configuration. For example, overoxidation of PPy and PANI typically occurs within the range of approximately +0.6 to +1.0 V (*vs.* Ag/AgCl), although this threshold may shift depending on the local microenvironment, especially under the alkaline conditions characteristic of chronic wounds.^52^ This process leads to disruption of π-conjugation, dopant expulsion and irreversible degradation of electrical properties. To mitigate these risks while maintaining therapeutic efficacy, rational materials design is required. The use of CPs such as PEDOT enables operation within safer voltage ranges due to its lower oxidation potential and higher electrochemical stability. Increasing capacitance through porous, nanostructured or hydrogel-based architectures allows charge delivery to occur predominantly *via* capacitive mechanisms. The architectures with high surface area and interconnected 3D networks enhance electric double-layer capacitance (EDLC) while reducing ion diffusion paths, minimizing parasitic reactions and thus preserving the electrochemical integrity of the electrode.^178^ From a stimulation perspective, the use of pulsed or alternating electrical inputs, as opposed to continuous stimulation, can further limit charge accumulation and electrochemical drift, thereby improving both electrochemical and biological safety.

These material and operational considerations directly underpin the selection of clinically relevant ES modalities. Externally programmed ES approaches, including direct current (DC), pulsed current (PC) and alternating current (AC), are widely used in clinical wound care and have been shown to promote cell migration, proliferation and regenerative gene expression, thereby promoting wound healing. To improve comparison across studies, a summary of ES parameters and their therapeutic efficacy is given in Table 2. Representative examples in wound-healing applications include PEDOT:PSS-based systems that operate predominantly within capacitive regimes, such as PEDOT:PSS membrane (PDMS composite) under low-voltage DC stimulation (100–300 mV),^179^ 3D printed PEDOT:PSS electrodes under PC stimulation (1.8–3.0 V, 10 Hz)^120^ and PEDOT:PSS/PVA–κ-carrageenan composite dressings (DC, ∼1.5 V).^180^ These systems enable effective charge delivery at the wound interface, thereby promoting key healing processes, including keratinocyte migration, gene expression, angiogenesis and tissue regeneration, while minimising faradaic side reactions such as ROS generation and biomolecular oxidation that could otherwise impair wound repair.

**Table 2:** Electrical stimulation parameters, electrochemical mechanisms and biological outcomes of CP-based wound dressings

CP	Type of current	Voltage/current density/frequency/duration	Charge injection mechanism	Electrochemical regime	Dominant interfacial processes	Biological outcomes	Safety considerations	Reference
PEDOT:PSS membrane (PDMS composite)	DC	100–300 mV; 5 min every 12 h	Mixed ionic–electronic conduction with volumetric capacitive charge injection	Predominantly capacitive (non-faradaic)	Ion redistribution within hydrated PEDOT:PSS network; electric-field-induced modulation of cell-substrate adhesion and ECM interactions	Accelerated keratinocyte cell sheet formation and detachment; enhanced cell viability, angiogenesis and re-epithelialisation	Operates within safe electrochemical window; minimal water electrolysis and ROS generation; low risk of protein oxidation and cell damage	179
3D printed PEDOT:PSS flexible electrode	PC	1.8–3.0 V; 10 Hz; 1 ms pulse width; 10 min per day	Mixed ionic–electronic conduction with volumetric capacitive charge storage	Predominantly capacitive with limited faradaic contribution	Ion transport within porous hydrogel; electric-field-mediated NIH3T3 cell migration; enhanced mass transport; antibacterial activity from quaternary ammonium-modified chitosan	Accelerated wound closure (∼99% by day 14); enhanced re-epithelialisation and collagen deposition	Porous hydrogel architecture buffers local pH and current distribution; capacitive-dominated behaviour limits ROS generation and faradaic reactions	120
PEDOT:PSS/PVA–κ-carrageenan 3D printed dressing	DC	∼1.5 V; 5–15 min per day	Mixed ionic–electronic conduction with capacitive-dominated charge storage	Predominantly capacitive	Electric-field-induced L929 cell migration (electrotaxis); enhanced interfacial charge transfer; synergistic antibacterial and anti-inflammatory effects	Accelerated wound healing; enhanced angiogenesis (increase CD31); reduced inflammation (decrease IL-6); improved haemostasis and tissue regeneration	Low-voltage operation and hydrogel matrix minimise pH gradients and ROS generation; reduced risk of biomolecule oxidation	180
PPy *in situ* polymerised hydrogel	AC	5 V	Redox-driven ion exchange	Mixed capacitive/faradaic	Polymer oxidation/reduction coupled with ion ingress/egress; electrochemical modulation of cell activity	∼84% Wound closure; enhanced fibroblast migration and gene expression	Elevated potentials may induce overoxidation (>∼0.6 V), ROS generation and local pH changes	181
		40 Hz						
		1 h per day (3 days)						
PEDOT:PSS/PVA hydrogel	PC	12 V	Capacitive charge injection with enhanced ionic transport *via* supramolecular network	Mixed regime with reduced faradaic contribution	Interfacial charge redistribution; enhanced ion mobility through hydrogen-bonded network	Accelerated wound closure (>80%), angiogenesis, antibacterial activity	High applied voltage but mitigated by pulsed mode and high capacitance; controlled ROS beneficial for antibacterial effect	182
		0.2 Hz						
		30 min						

**Fig. 4:**
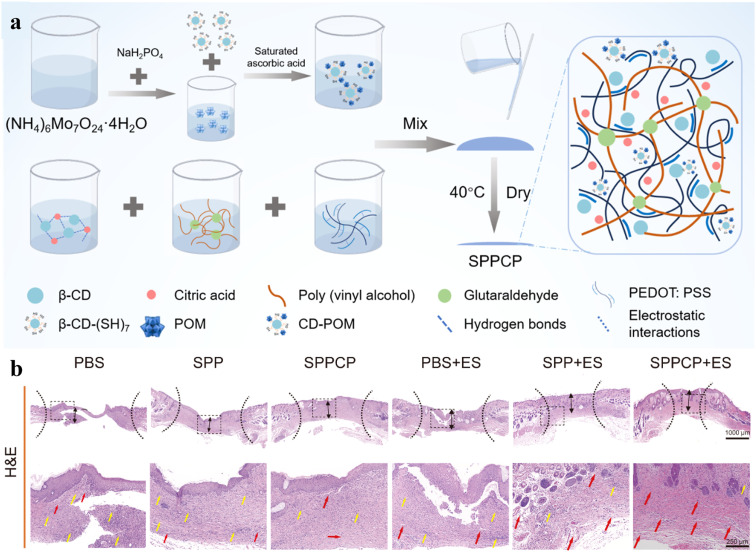
(a) Schematic of the preparation of SPPCP. (b) Treatment of bacteria-infected mice wound by SPPCP gels with ES. Hematoxylin and Eosin (H&E) staining was used to observe tissue healing (yellow arrow: inflammatory cells; red arrow: blood vessels). Adapted with permission from ref. 182 Copyright © 2025 Elsevier.

When ES is delivered through CP-based dressings, the healing outcomes are enhanced due to more consistent stimulation delivery and uniform electric field distribution.^6^ For example, PPy-based hydrogel (conductivity around 0.5–0.8 mS cm^−1^) was placed over fibroblast cell voids.^181^ ES (AC at 5 V and 40 Hz for 1 h, 3 days) *via* the PPy hydrogel enhanced the scratch closure effectively with an average of 84% void reduction after 12 h, while ES through the silver electrode filled 77% of the void. Upregulation of fibroblast-associated genes involved in migration was observed. In another study, a PEDOT:PSS/PVA hydrogel incorporated a citric acid–β-cyclodextrin (β-CD) supramolecular system and cyclodextrin–polyoxometalates (CD–POM), termed SPPCP, was developed for complex bacteria-infected wound management (Fig. 4).^182^ The high conductivity of the hydrogel, approximately 20 S cm^−1^, was attributed to the strong hydrogen bonding interactions between abundant carboxyl and hydroxyl groups within the supramolecular system, forming a supramolecular network, as well as the supramolecular system interacting with positively charged PEDOT *via* electrostatic interactions.^183^ Upon application of ES (PC 12 V, 0.2 Hz, 30 min), the dressing exhibited significantly enhanced antibacterial activity and further stimulated fibroblast migration and angiogenesis. *In vivo*, ES delivered *via* the SPPCP accelerated wound closure to over 80% within 10 days of mice infectious wound model, while simultaneously reducing tissue inflammation and promoting collagen deposition more effectively than ES delivered through metal electrode.

ES-enabled by CPs may exhibit synergetic antibacterial activity, making them particularly suitable for the treatment of infected wounds.^184^ The antibacterial mechanisms of ES mainly include disrupting the balance of bacterial cell membrane potential, increasing membrane permeability and interfering with electron transfer in bacteria to increase ROS production.^185–188^

In summary, electrical stimulation offers an effective means to restore disrupted endogenous bioelectric cues in chronic and non-healing wounds, thereby enhancing cell migration, angiogenesis and tissue regeneration. CP-based wound dressings provide clear advantages over conventional metal electrodes for ES by enabling mechanically compliant, low impedance and low-voltage stimulation, while also offering synergistic antimicrobial functionality. However, challenges remain in fixed, pre-determined stimulation parameters and the lack of integrated real-time wound monitoring, limiting its ability to dynamically adapt therapy to the evolving wound microenvironment.

## 4. Electrochemical drug release from CP-based dressings

CPs have gained significant interest in drug-delivery applications due to the low electrical potential that can be used to control the expulsion of molecules bound within CPs through doping.^10,34,189^ On-demand drug delivery is essential in wound healing because wound microenvironments and therapeutic needs change dynamically over time. The utilization of CPs as a dressing for a programmable drug delivery system is a promising approach to satisfy this major need. CPs possess substantial porosity and delocalised charge centers to allow counter-ion diffusion and electromigration inside the polymeric electrode body in response to oxidation or reduction.^42,190^ In electrically-triggered release mechanisms, CP films are used to deliver or gate the movement of specific molecules, usually ions, on demand and in a programmed manner.^42^

The reversible redox behaviour of CPs enables the loading of wound healing drugs in an electrochemically controlled manner, including *via* electropolymerisation, physical retention and by covalent binding.^191^ In 1984, Zinger *et al.*^192^ achieved the first electrochemical delivery of a bioactive molecule, glutamate, from PPy film, where glutamate and ferrocyanide were used as dopants within the CPs matrix. That study established the foundation for CP-based electroresponsive drug delivery systems. Nowadays, it is well-known that the controlled release of therapeutics from CP matrices is driven by the redox properties of CPs with an externally applied electrical potential. Importantly, the release behaviour is not universal but strongly dependent on the physicochemical properties of the drug, particularly charge, molecular weight and hydrophilicity/hydrophobicity, which govern both loading efficiency and electrochemically triggered release kinetics across different therapeutic classes (*e.g.*, antibiotics, anti-inflammatory agents and growth factors).

**Fig. 5:**
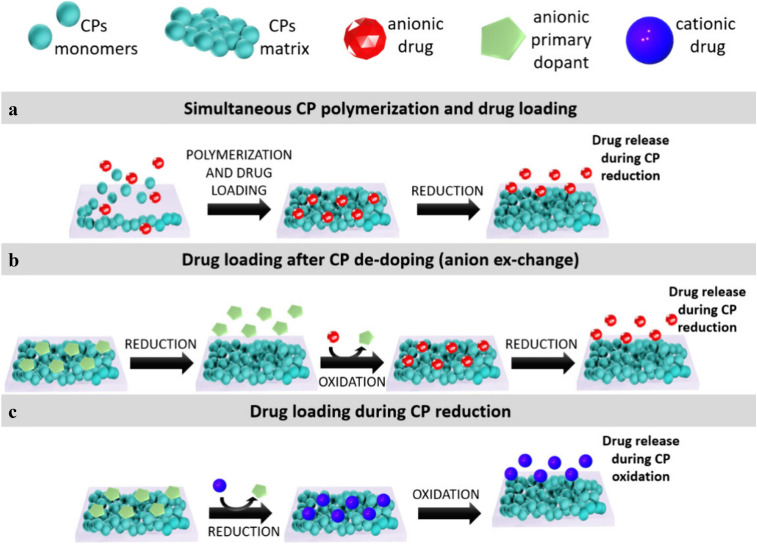
General mechanisms for drug loading into CPs matrices: (a) One-step loading of drugs during CPs polymerization, (b) loading of drugs after CPs polymerization by anion exchange and (c) loading of cationic drugs during CP reduction. Adapted with permission from ref. 189. Copyright © 2024, *ACS Applied Polymer Materials*.

Therapeutic loading into CP-matrices can be conducted in four different ways (Fig. 5), depending on the main features of the drug, such as charge (anionic, cationic or neutral) and physicochemical properties:^189,193^ (i) direct incorporation of anionic drugs as counter-ions during CP electropolymerisation (Fig. 5a), (ii) post-polymerisation loading of anionic drugs *via* redox-mediated ion exchange (Fig. 5b), (iii) post-polymerisation loading of cationic drugs upon reduction of CPs (Fig. 5c), typically facilitated by the presence of large immobile anions used as dopants for CP synthesis, and (iv) immobilisation through entrapment, impregnation or adsorption. Method (iii) allows various therapeutic agents for wound healing to be loaded if they are charged and has been used to successfully load, *e.g.* curcumin, dexamethasone phosphate, growth factors, heparin, adenosine triphosphate (ATP) and chitosan in CPs.^194–198^ Method (iv) is a useful strategy that allows biomolecules to be incorporated without undergoing a chemical process that can alter their activity.^199,200^ Biomolecules, like DNA and proteins, can be incorporated into CPs *via* physical retention.^189^

Notably, anionic drugs generally exhibit higher loading efficiency due to strong electrostatic interactions with oxidised CP backbones, whereas cationic or neutral molecules are more often incorporated *via* physical entrapment, resulting in comparatively lower loading efficiency and less controllable release behaviour.^201^

The redox processes of CPs produce volumetric changes through an electro-chemo-mechanical response.^202–204^ This processes enable the expulsion of bioactive molecules from the CPs-matrix. For instance, the electrochemical reduction of PPy hydrogel led to volumetric contraction that actively facilitated the desorption of curcumin NPs from the PPy.^205^ As a result, an approximately twofold increase in both the curcumin release rate and overall release efficiency was achieved compared with passive, non-stimulated controls. The enhanced retention and subsequently improved release kinetics are heavily influenced by molecular hydrophobicity. Hydrophobic compounds like curcumin form strong interactions with the CPs backbone, making active actuation (*e.g.*, *via* ES) necessary for efficient release.^34^

**Fig. 6:**
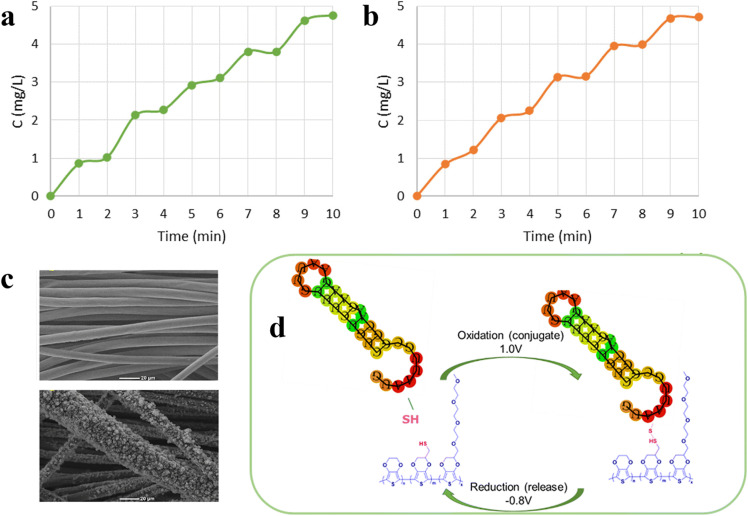
ON/OFF release of ibuprofen: drug is released when a positive voltage is applied, while retention is observed at −0.3 V. (a) PPy, PEDOT mix rim system prototype; (b) PPy single system prototype. Adapted with permission from ref. 206 Copyright © 2023 MDPI. (c) SEM images of uncoated carbon cloth (CC) (top) and EDOT-*co*-EDOTSAc-*co*-EDOTEG coated CC (bottom). (d) Electrochemical coupling of thiol-functionalised connexin43 through the oxidative formation of disulfide and cleavage by reduction of the disulfide, on EDOT-*co*-EDOTSAc-*co*-EDOTEG coated CC substrates. Adapted with permission from ref. 114 Copyright © 2023 *RSC Applied Polymers*.

In addition to electro-chemo-mechanical actuation, CP-based dressings enable voltage-controlled drug release *via* redox-mediated electrostatic interactions and ion transport. Small, anionic drugs (*e.g.*, anti-inflammatory agents) are particularly well-suited for rapid and reversible electrostatic (redox-driven) release. For example, an electrospun membrane composed of cellulose acetate (CA) and ibuprofen (IBU), followed by electrochemical deposition of PEDOT and PPy, enabled electrochemical retention and release of the anionic drug ibuprofen through redox-dependent electrostatic interactions.^206^ The application of a negative potential (−0.3 V) retained the IBU within the matrices, whereas positive potentials (+0.3 to +0.8 V) significantly accelerated release, achieving a reversible ON/OFF release profile (Fig. 6a and b). Such controllable delivery prevents the rapid degradation of biological substances like nerve growth factors^207^ and maintains therapeutic concentrations at the wound bed.

Similarly, Kleber *et al.*^208^ reported pulsatile release of anionic actives, like dexamethasone (Dex) and fluorescein sodium salt, from poly(dimethylacrylamide-*co*-4-methacryloyloxy benzophenone-*co*-4-styrenesulfonate) (PDMAAp)/PEDOT hydrogel networks. In this system, drug release was governed by electrostatic interactions between the anionic drugs and the oxidised PEDOT matrix. When a negative potential of −0.5 V was applied for 60 s, a burst release of around 120 ng was recorded, whereas cyclic voltammetry stimulation (−0.5 to 0.8 V at a scan rate of 0.1 V s^−1^) generated sustained and stepwise release profiles by repeatedly modulating the polymer redox state. Such behaviour suggests that electrostatic (redox-driven) release dominates for small, charged molecules, whereas the redox-driven osmotic expansion mechanism may become more significant for larger or weakly charged therapeutics.^209^

Beyond single-drug systems, voltage-selective and multi-drug release has been achieved using PEDOT films doped with drug-loaded silica nanoparticles (SNPs),^210^ in which oppositely charged model compounds, doxorubicin (Dox) and melatonin, were encapsulated within the mesoporous SNPs to enable electrically addressable and selective release. Here, cyclic voltage was swept between 0.8 V and −0.6 V, effectively triggering Dox elution, achieving a cumulative release of 7.4 µg cm^2^ over 200 stimulations. In contrast, melatonin release occurred only at lower applied potentials (<0.3 V). This behaviour may be attributed to the differences in redox-driven charge compensation within the PEDOT matrix. The lower potential likely decreased the total positive charge on the PEDOT, weakening electrostatic interactions and facilitating the release of weakly charged or neutral molecules such as melatonin. Furthermore, the differences in charge state and size of the drug molecules (melatonin only adopts a mild positive charge, whereas Dox was used in its salt form, and with molecular weights of 232 g mol^−1^ and 534 g mol^−1^, respectively) likely influence diffusion kinetics and release at which the compound exits the film. Together, these findings demonstrate that distinct therapeutic agents can be released independently with tunable kinetics from a single system, where release selectivity is governed by a combination of molecular weight, charge and hydrophilicity, providing a rational basis for designing CP-based platforms for tailored multi-therapeutic delivery.

Covalent binding of biomolecules to the CPs is another approach that enables long-term stability and guarantees that the biomolecules are firmly linked and will not be released by diffusion.^211^ This strategy is particularly advantageous for large biomolecules (*e.g.*, growth factors or nucleic acids) and neutral therapeutic agents. Recently, Beikzadeh *et al.* reported a switchable, electrochemically controlled disulfide bridge linker reduction strategy to regulate drug (connexin43) loading and release in CP-based dressings.^114^ In this system, a wound-healing therapeutic oligonucleotide drug was electrochemically conjugated to a thiol-functionalised PEDOT copolymer-coated carbon cloth and an electrospun fiber mat surface under an applied oxidative potential (+1.0 V) *via* disulfide bond formation. Subsequent application of a reductive potential (−0.8 V) was used to cleave the disulfide linkage, enabling on-demand electrochemical release of the conjugated drug (Fig. 6c and d).

A wide range of therapeutics in wound healing applications has been electrochemically loaded into the CPs-based dressings, including antibiotics,^212^ anti-inflammatories,^213^ anti-oxidants^214^ and pro-regenerative drugs, as well as bioactive molecules such as growth factors, cytokines and peptides.^215^

CPs-based dressings with drug delivery function can be specifically designed to target distinct stages of the wound healing process, thereby enhancing both biocompatibility and functionality. Sirivisoot *et al.* developed nanostructured PPy coatings, deposited electrochemically onto commercially pure titanium, incorporating either antibiotic (penicillin/streptomycin, P/S) or an anti-inflammatory agent (dexamethasone, Dex).^216^ Upon five electrochemical redox cycles in the range between −1 V and 1 V, approximately 80% of the initially incorporated drugs were released. The P/S-loaded PPy coatings exhibited pronounced bactericidal activity against *Staphylococcus epidermidis* within 1 h. Furthermore, the drug-loaded PPy layers showed significantly reduced bacterial colonisation and macrophage adhesion. Overall, these results highlight that CP-based drug delivery systems must be tailored according to drug-specific physicochemical properties to optimise release kinetics, therapeutic efficacy and stage-specific wound healing outcomes.

## 5. CP-based sensors for wound healing

A wide range of biomarkers associated with wound status and infection have been identified, reflecting the complex biochemical and physiological processes occurring at the wound interface. These biomarkers can be broadly classified into physicochemical parameters (*e.g.*, pH and temperature), metabolites (*e.g.*, uric acid, lactate), signalling molecules (*e.g.*, nitric oxide (NO), hydrogen peroxide (H_2_O_2_), cytokines) and bacterial-derived products.^217–220^

Among these, wound pH, temperature and uric acid concentration are the most extensively studied indicators due to their strong correlation with infection, inflammation and delayed healing. The normal pH of skin or healing wounds is acidic (5.5–6.5), while chronic wounds or infected wounds with high bacterial load often have an alkaline pH above 7.3.^221^ Meanwhile, wounds with elevated temperature are eight times more likely to be infected by bacteria.^221^ Moreover, uric acid concentration in wound exudate, associated with the colonization of *Staphylococcus aureus* or *Pseudomonas aeruginosa*, also have strong correlation with wound severity.^222^ Given the dynamic and heterogeneous nature of chronic wound microenvironments, there is a growing trend toward the development of smart wound dressings capable of simultaneously monitoring multiple biomarkers, enabling more comprehensive and real-time assessment of wound status.^223^

CPs provide a particularly attractive platform for wound biomarker sensing owing to their mixed ionic–electronic conductivity, redox activity and tunable interfacial functionalization. These properties allow CPs to convert biochemical interactions into electrical signals, enabling continuous, *in situ* monitoring of dynamic changes within the wound microenvironment.^224,225^ CP-based electrochemical sensors operate through potentiometric, amperometric, conductometric or impedimetric modes.^226,227^ Their sensitivity is often governed by interfacial charge transfer, ion transport and surface functionalization. In the next paragraphs, some prominent examples of electrochemical CP-based sensing for the detection of wound-relevant biomarkers are discussed. Detailed reviews on electrochemical CP sensors can be found in recent literature,^227,228^ as well as on CP-based organic electrochemical transistor (OECT) sensors.^148,229^

**Fig. 7:**
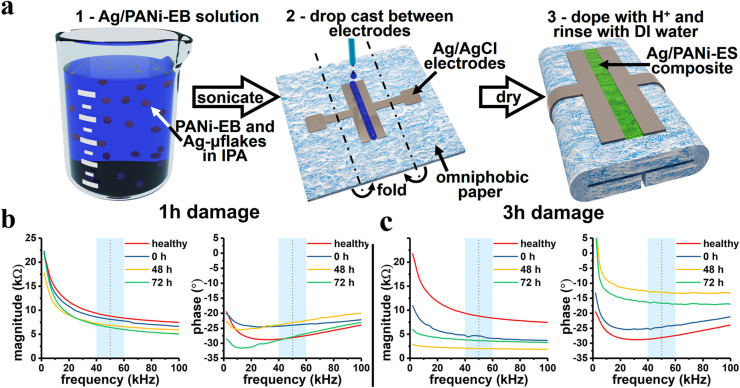
Schematic diagram describing the fabrication of Ag/PANI composite electrodes. (a) Early *in vivo* detection and monitoring of pressure-induced tissue damage using OPSB (b and c). Bode diagrams of the magnitude and phase of the impedance measured across pressure ulcer models induced on a mouse by 1 h (b) and 3 h (c) of ischaemia cycles. Adapted with permission from ref. 231 Copyright © 2018 Elsevier.

PANI undergoes reversible transitions between distinct oxidation and protonation (doping) states in response to pH changes, leading to measurable variations in electrical resistance, making it well-suited for pH sensing.^230^ An omniphobic paper-based smart bandage (OPSB) for the simultaneous electrochemical detection of uric acid (UA) and wound pH was reported by Pal *et al.*^231^ The selected electrodes were screen-printed onto omniphobic paper and incorporated into commercially available bandages. As shown in Fig. 7, two silver electrodes were printed, and a layer of PANI was deposited between them. UA was detected using uricase-modified electrodes using amperometry. For pH measurements, electrochemical impedance spectroscopy (EIS) was used. To demonstrate practical utility of the OPSB, the OPSB was successfully adapted for tissue impedance measurements for the early detection of pressure ulcers in mice.

Also, a smart bandage was fabricated by depositing PEDOT:PSS over a 2 mm gap between two Ag ink electrodes over a commercial poly(vinyl chloride) (PVC) substrate for temperature sensing.^232^ The PEDOT:PSS-based temperature sensor showed a ∼70% decrease in resistance for a temperature change from 25 °C to 90 °C with a sensitivity of ∼1.2%/°C. This thermosensitive behaviour arises from the temperature-dependent charge transport characteristics of PEDOT:PSS.^233^ To enhance the stability under humid conditions and temperature sensitivity of the sensor, Wang *et al.*^234^ employed (3-glycidyloxypropyl) trimethoxy silane (GOPS) to crosslink the hydrophilic PSS in PEDOT:PSS. The resulting sensor exhibited stable performance over a wide humidity range (30–80% RH), high thermal sensitivity (−0.77%/°C from 25 to 50 °C) and excellent mechanical robustness.

To improve sensitivity toward wound-relevant biomarkers, a range of CP-based composites have been developed to reduce the limit of detection by enhancing electrical conductivity and effective surface area. For example, PPy-based composites incorporating conductive or catalytic components, such as metal–Au,^235^ metal oxides–ZnO^236^ and graphene oxide^237^ or carbon nanomaterials,^238^ have been shown to facilitate efficient electron transfer and improve the electrochemical detection of metabolites associated with wound inflammation and infection. In particular, PPy-based composite electrodes have demonstrated sensitive detection of wound-relevant analytes, including lactate,^238^ small-molecule drugs, ibuprofen^239^ and T cells,^237^ by introducing abundant active sites, enhancing interaction and recognition efficiency.

**Fig. 8:**
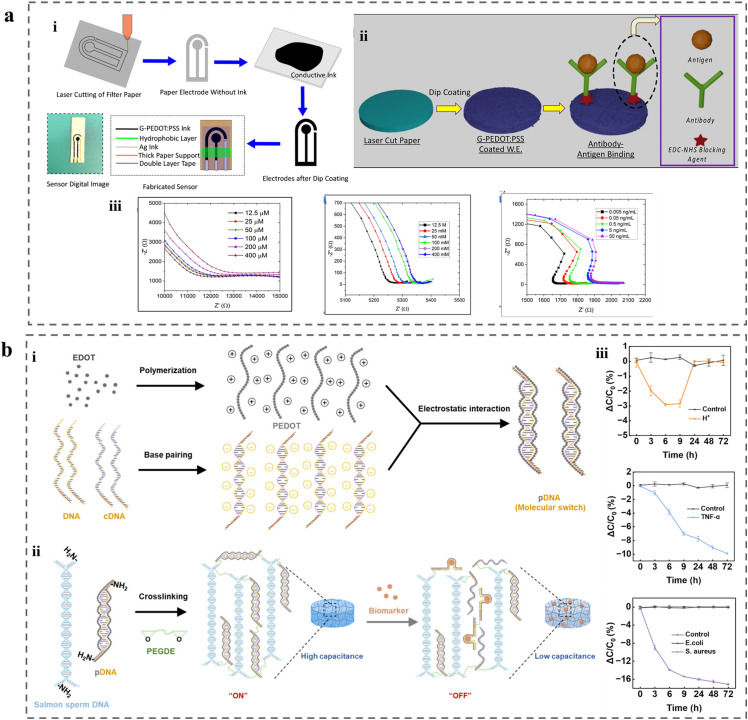
(a) Paper-based biosensor fabrication and detection of dopamine, TNF-α and IL-6 *via* EIS. (i) Fabrication and (ii) probe proteins immobilization. (iii) EIS detection of dopamine (left), TNF-α (middle) and IL-6 (right). Adapted with permission from ref. 240 Copyright © 2023 MDPI. (b) Preparation of DNA hydrogel capacitive sensor and the capacitive monitoring in diabetic wounds. (i) Electrostatic assembly of PEDOT polymer and partially complementary DNA duplex. (ii) Crosslinking of pDNA, salmon sperm DNA and PEGDE into hydrogel networks. Biomarker recognition triggers pDNA duplex dissociation, reducing hydrogel capacitance. (iii) Relative capacitance changes over time upon exposure to target stimuli of H^+^ (top), TNF-α (middle), and bacterium (bottom). Adapted with permission from ref. 242 Copyright © 2025 American Chemical Society.

CP-based sensors have been extended toward inflammatory biomarkers to address the need for monitoring of immune responses during wound healing. A representative example used a graphene/PEDOT:PSS composite working electrode in an electrochemical biosensor designed for *in situ* monitoring of wound inflammation^240^ (Fig. 8a). In this system, dopamine was detected directly *via* its electrochemical oxidation, while pro-inflammatory cytokines, including tumour necrosis factor-α (TNF-α) and interleukin-6 (IL-6), were detected using immobilised antibody-based receptors. Electrochemical characterisation using cyclic voltammetry and electrochemical impedance spectroscopy enabled quantitative detection of dopamine (12.5–400 µM) in PBS, TNF-α (0.005–50 ng mL^−1^) and IL-6 (2 pg mL^−1^–2 µg mL^−1^), achieving low limits of detection of 3.4 µM, 5.97 pg mL^−1^ and 9.55 pg mL^−1^, respectively. The antibody-functionalised sensors exhibited high selectivity against interfering proteins (*e.g.*, serpin A1) and successfully detected IL-6 in human serum, demonstrating the sensor's applicability for monitoring inflammatory responses in wound environments.^241^

A highly sensitive and rapid-response PEDOT:DNA (pDNA) hydrogel sensor was developed to address pH, inflammation and infection dynamics in diabetic wound management (Fig. 8b).^242^ In this system, PEDOT chains were polymerised along DNA duplexes, forming pDNA complexes. Subsequent cross-linking was achieved *via* nucleophilic ring-opening reactions between primary amine groups on unpaired pDNA bases and epoxy groups in poly(ethylene glycol) diglycidyl ether (PEGDE), yielding a conductive hydrogel network. Within the hydrogel, pDNA comprises a metastable DNA duplex formed by a programmable biomarker-responsive nucleic acid strand and a partially complementary strand. Upon encountering stimuli (H^+^, TNF-α and bacterium), the responsive sequence undergoes a conformational change into an i-motif or aptamer structure. This dissociates the pDNA duplex and disrupts the conductive network, resulting in a measurable change in hydrogel capacitance. The hydrogel sensor exhibited sensitive capacitive responses across clinically relevant ranges of pH (7.0–5.0), TNF-α (0.3–2.5 pM) and *Staphylococcus aureus* (1 × 10^3^–10^13^ CFU mL^−1^).

CPs also serve as the active channel materials in organic electrochemical transistors (OECTs).^243–245^ OECTs can be integrated with flexible and textile substrates, allowing conformal contact with soft tissues and wound beds. For example, a PEDOT:PSS-based OECT was fabricated directly onto medical gauze *via* screen printing.^246^ The device continuously absorbed wound exudate for potentiostatic detection of UA (detection range of 220–750 µM in synthetic wound exudate).

Oxygen sensing is particularly relevant to wound healing, as local oxygen availability regulates cell proliferation, angiogenesis and antimicrobial defense.^247^ An OECT-based oxygen sensor employing PEDOT:PSS channels, hydrogel electrolytes and oxygen-permeable membranes (polydimethylsiloxane, PDMS).^248^ The oxygen-sensing mechanism relies here on electron transfer from the PEDOT:PSS to the oxygen, which generates hole carriers in the PEDOT phase and amplifies the transistor current, with oxygen reduced to hydrogen peroxide. The sensor device achieved a fast response (93.2 ± 0.8) µs.

These above discussed studies underscore the strong potential of CP-based electrochemical and OECT-based sensors to enable sensitive, selective and *in situ* monitoring of key physicochemical and biochemical parameters associated with wound healing and infection. By transforming passive dressings into active diagnostic interfaces, these sensing platforms can provide critical insights into the dynamic wound microenvironment. However, despite their diagnostic capability, the majority of CP-based sensors currently operate as standalone monitoring tools, with limited capacity to actively intervene in the wound-healing process. This separation between sensing and therapy highlights an important gap between wound-state assessment and therapeutic decision-making.

Another critical limitation lies in the long-term stability of CP-based sensors under complex wound conditions. CP-based sensors operating in wound environments are highly susceptible to biofouling and signal drift, which significantly compromise long-term reliability. Protein adsorption, bacterial colonisation and accumulation of extracellular matrix components can progressively block the binding sites or alter the local ionic and pH environment, leading to false signals or drift.^249^ In addition, continuous redox cycling of CPs may induce structural rearrangement, dopant loss or overoxidation, further contributing to signal instability during prolonged operation.^250^ To mitigate these challenges, several chemical and interfacial engineering strategies have been explored. One widely adopted approach involves the incorporation of antifouling coatings, such as zwitterionic polymers (*e.g.*, sulfobetaines, carboxybetaines, peptides) anchored to CPs,^251,252^ which form strong hydration layers *via* electrostatically induced water structuring, thereby resisting nonspecific protein adsorption. Wu *et al.*, reported a zwitterionic poly(sulfobetaine-3,4-ethylenedioxythiophene) (PSBEDOT) glucose biosensor which showed good stability in 100% human blood plasma, with the current signal remaining over 90% after the sensor being stored in human blood plasma for 14 days.^252^ Similarly, hydrophilic polymer brushes or PEG-based coatings reduce biofouling through steric repulsion and hydration effects. A biosensor based on the PEGylated PANI nanofibers supported the quantification of DNA in complex human serum, and it retained approximately 92.14% of its original signal after 10 days.^253^

Integration of hydrogels has been demonstrated to enhance interfacial stability and mitigate signal drift in CP-based sensors including PEDOT:PSS-based or composite hydrogels. Such systems provide a hydrated, permeable matrix that facilitated ion transport while physically limiting the adsorption of macromolecules and cells.^35,254^ These hydrogel layers can also buffer mechanical mismatch and stabilise the electrode–tissue interface, thereby reducing signal fluctuation under dynamic wound conditions. Protective membranes, such as selectively permeable polyurethane or polydimethylsiloxane (PDMS) layers, have also been employed to act as a diffusion barrier, allowing small analytes (*e.g.*, oxygen, uric acid) to reach the sensing interface while excluding larger fouling species.^247^ Despite these advances, long-term operational stability at clinically relevant timeframes (>14 days) remains insufficiently understood. Progressive biofouling, biofilm formation and electrochemical drift continue to degrade sensor performance, while antifouling strategies often introduce trade-offs in sensitivity and response time due to additional diffusion barriers.^249^ Therefore, future development of CP-based wound sensors should include rational designs of multifunctional interfaces that integrate antifouling capability, electrochemical stability and selective permeability, enabling reliable and sustained monitoring in complex wound environments.

## 6. Next-generation CP-based wound dressings

**Fig. 9:**
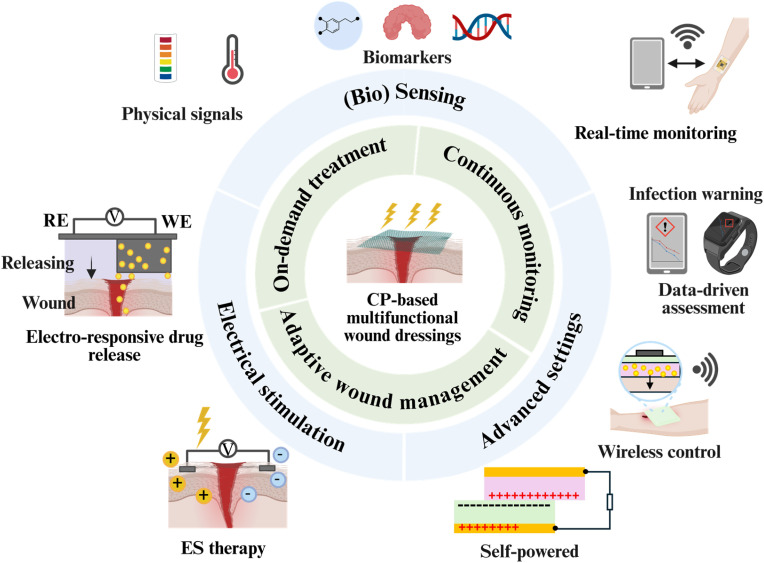
Schematic of multifunctional CP-based wound dressings that combine on-demand treatment, continuous monitoring and adaptive wound management, towards closed-loop wound care. Created in BioRender. Yang, J. (https://BioRender.com/a4tlhw5) is licensed under CC BY 4.0.

Although CP-based wound dressings have demonstrated therapeutic efficacy separately using strategies of electrical stimulation therapy, drug delivery or wound monitoring, single-function systems typically address only one dimension of the highly complex wound-healing cascade, thereby overlooking critical interdependencies between infection control, inflammation regulation, angiogenesis and tissue regeneration.^255^ For instance, while ES alone can enhance cell migration and angiogenesis, inappropriate current density or prolonged stimulation may pose risks without controlled intensity and duration guided by wound biomarkers measurements.^256^ Similarly, standalone drug delivery platforms generally lack the capacity to dynamically modulate release in response to the evolving wound microenvironment.^257^ In addition, externally programmed ES restricts device portability, long-term wearability and patient compliance. These limitations collectively motivate the development of next-generation CP-based wound dressings that integrate on-demand treatment, continuous monitoring and adaptive wound management within a unified platform, enabling intelligent, personalised, closed-loop wound care (Fig. 9).

The combination of ES and drug delivery in CP-based wound dressings enables on-demand treatment, while further integration of CP-based sensors allows real-time monitoring of wound biomarkers and the precise regulation of drug release and/or ES.^258^ Representative examples of such multifunctional CP-based wound dressings, along with the key features and limitations, are summarised in Table 3.

**Table 3:** Examples of multifunctional CP-based wound dressings summarising key features and limitations

System^[reference]^	Fabrication	Conductivity	Integrated functions	Energy source	Key therapeutic outcomes	Key limitations
Heparin-doped PPy/PLA membrane^259^	Electrochemical PPy deposition and heparin doping	N/A	ES + electro-responsive drug release	External power supply	Increased fibroblast activity, FGF-1/FGF-2; accelerated myofibroblast trans differentiation	Requires external power; risk of over-/under-stimulation; redox fatigue and dopant leaching
Vitamin D-loaded PANI/CS hydrogel^260^	Ionic gelation of chitosan (tripolyphosphate) with dispersed PANI and vitamin D	N/A	ES + growth-factor delivery	External stimulator	Faster healing (12 *vs.* 21 days); reduced scarring; enhanced re-epithelialization	Non-adaptive ES parameters: diffusional drug release not wound-responsive
PDA-doped CP/nanozyme hydrogels (PDA@PPy; PDA-Fe-PEDOT)^261,262^	*In situ* CP polymerisation with PDA/nanozyme incorporation into chemically crosslinked hydrogel network	0.01 to 0.04 S cm^−1^ (PDA@PPy)	ES support + ROS scavenging + inflammation regulation	External power	Improved collagen deposition and angiogenesis in diabetic/infected wounds	System complexity; reproducibility; unclear long-term degradation and redox stability
GelMA/PPEDOT NPs/DA/LBP	Chemical polymerisation of PPEDOT NPs	14 S m^−1^	ES responsiveness + immunomodulation + antioxidant activity + electro responsive LBP release	External stimulator	Decreased inflammation (TNF-α, IL-6); M1 → M2 macrophage polarization; increased collagen deposition, angiogenesis and peripheral nerve regeneration	Conductive NPs have a low dispersibility; physical coupled system
Microneedle patch	UV crosslinked to form the needle patch, and combined by sequential assembly					
Self-assembled flexible 3D array on LIG^151^	LIG patterning with sequential electrochemical deposition of CPs and LbL assembly	N/A	pH sensing + UA sensing + antibiotic release	External circuit	N/A	Signal drift; circuit integration and circuit integration complexity
QOSP electret-inspired hydrogel^269^	Schiff-base hydrogel formation with embedded PANI nanowires followed by plasma charge injection	3.33 × 10^−5^ S m^−1^	ES + antibacterial + immunomodulation	Stored electrostatic charge	Accelerated burn wound healing; decreased fibrosis; immune reprogramming (Th2 shifted to Th1)	Surface potential decay over time; dielectric constant and loss are observed to increase with temperature
Zn-PEDOT PIT battery hydrogel patch^270^	*In situ* polymerisation of ion–electron dual-conductive hydrogel coupled to Zn anode	33.2 S m^−1^	ES + electrophysiological sensing + antibacterial	Zn anode	Tunable antibacterial microcurrents; promoted angiogenesis and collagen deposition	Limited cycling stability (52% capacity after 10 cycles); metal ion management
Triboelectric stimulator + PPy hydrogel^271^	Microstructured flexible TENG fabrication integrated with electro-polymerised PPy drug-loaded hydrogel	N/A	ES + mechano-triggered drug delivery	TENG, patient motion	Promoted cell migration; decreased inflammation (IL-1, TNF-α, IL-6); enhanced infected-wound healing	Motion-dependent output; inconsistent current; patient-activity reliance
PPy@PDA/PANI hydrogel + PANI/PVDF film^153^	*In situ* polymerisation of PPy@PDA nanowires within a PANI hydrogel, integrated with a PANI/PVDF flexible film	N/A	Real-time ammonia sensing + ES + drug release + closed-loop feedback	External power supply with wireless control	High-sensitivity NH_3_ sensing (LOD ∼49 ppt); precise electrically triggered drug release; effective infected wound management *via* closed-loop control	Dependence on external power and wireless hardware; system complexity may limit scalability and long-term clinical robustness
PEDOT:PSS adhesive hydrogel on FPCB smart bandage^272^	PEDOT:PSS-based adhesive hydrogel interfaced with a flexible printed circuit board incorporating sensors, stimulation circuits, and RF components	N/A	ES + continuous monitoring of wound impedance and temperature + closed-loop feedback	Wireless inductive coupling *via* RFID/NFC	Continuous wound-state monitoring, ∼25% faster wound closure and ∼50% enhanced dermal remodelling in mouse models	Reliance on external RFID reader; limited sensing modalities; long-term stability and translation to human wounds remain to be demonstrated

An example of dual-function CP-based wound healing platform, that couple drug release and ES, is a heparin-doped PPy/PLA conductive membrane.^259^ This system enabled electrochemical release of heparin upon reduction of the PPy, while applying ES resulting in enhanced fibroblast activity, elevated fibroblast growth factors 1 (FGF-1)/FGF-2 expression and accelerated myofibroblast differentiation. In another example, vitamin D loaded PANI/chitosan (CS) hydrogels exploited PANI redox switching and ionic crosslinking to couple ES with vitamin D delivery achieving complete wound closure on rats' model within 12 days *in vivo*, compared with 21 days for untreated controls.^260^

More recently, polydopamine (PDA)-modified CP nanozyme hydrogels have integrated ES with redox-active nanozyme functionality to scavenge excess reactive oxygen species and modulate inflammation in diabetic wounds. The PDA@PPy-based hydrogel composed of a framework of hyaluronic acid (HA) modified with phenylboronic acid (PBA) and ε-polylysine (EPL) linked to caffeic acid, designed to release nanoenzyme in response to changes in ROS and pH levels. The incorporation of PDA@PPy into the hydrogel matrix increased the conductivity and aided in inflammation control through ES, and guaranteed a steady supply of O_2_.^261^ Another study employed dopamine-mediated PEDOT (PDA-Fe-PEDOT) nanozymes within dopamine-grafted fish gelatin with methacrylated silk fibroin hydrogel framework.^262^ The PDA-Fe-PEDOT was synthesised *via* DA-mediated polymerisation of EDOT under FeCl_3_-induced oxidation. The system is capable of catalysing exogenous H_2_O_2_ to generate hydroxyl radicals (˙OH), offering potent antibacterial activity to prevent wound infection. Meanwhile, the DA-rich hydrogel exhibited strong antioxidant capacity, effectively scavenging excess ROS at the wound site. This effect is attributed to the oxidation of phenolic hydroxyl groups in DA to quinones during ROS scavenging. Importantly, ES application enabled the reduction of quinone groups in DA back to phenolic hydroxyl groups, thereby partially restoring and extending the hydrogel's antioxidant functionality.^262^ Recent research suggests that polyphenols, when combined with CPs, can form electron donor–acceptor complexes that help maintain the redox balance between catechol and quinone groups, thereby preserving their antioxidant functionality.^263^

To address infection in deeper tissue layers, CPs were integrated into microneedle systems (MNs) for minimally invasive delivery and localised electrotherapy.^264–266^ Hou *et al.* reported a hydrogel MNs comprised of GelMA, PDA-modified PEDOT NPs (PPEDOT), dopamine (DA) and lycium barbarum polysaccharide (LBP).^267^ PPEDOT imparted electrical conductivity to the MNs, enabling modulation of the wound microenvironment through ES. The conversion of catechol–quinone through electron transfer in response to ES facilitated the release of LBP, which, in combination with ES, promoted regeneration of wound tissues and peripheral nerves. Valdés-Ramírez *et al.* further proposed an electrochemically switchable nanoactuator that was capable of delivering multiple therapeutic agents.^268^ Two individually addressable channels based on PPy/dodecylbenzene sulfonate (DBS) on a single MN array, enabled programmable ON–OFF and multiplexed delivery of distinct model compounds (dyes). Although this system has not yet been applied to chronic wound healing, it holds significant potential for such applications, particularly for the co-delivery of multiple wound-healing therapeutics.

Multilayer CP-based dressings have also been developed to integrate sensing and therapy within a single device. A self-assembled 3D patch based on PANI, PPy and PEDOT integrated sensing and on-demand antibiotic release.^151^ Specifically, the pH sensor was comprised of a laser-induced graphene (LIG) working electrode modified with a PANI layer (PANI/LIG). The UA sensor was fabricated from PEDOT embedded with Prussian blue composite (PEDOT:PB) on LIG *via* a facile one-pot electrochemical deposition, followed by deposition of uricase onto the PEDOT:PB/LIG through physical entrapment. The drug delivery was achieved *via* an electrically triggered drug release of ciprofloxacin (Cipro) from a PPy:Cipro/LIG patch. Consequently, this integrated smart bandage platform enabled electrochemical measurements of pH levels (over the range of 4–10) and UA concentrations (up to 0.9 mM), as indicators of wound status, while also facilitating on-demand release of Cipro *via* +0.6 V ES as needed based on pH/UA monitoring measurements.

Most multifunctional CP-based wound dressings reported to date rely on external power sources and externally programmed ES, which enables precise control over stimulation parameters but limits device portability and increases system complexity. In contrast, self-powered wound dressings integrate energy generation or storage directly within the dressing, allowing autonomous electrical functionality without continuous external power input.

**Fig. 10:**
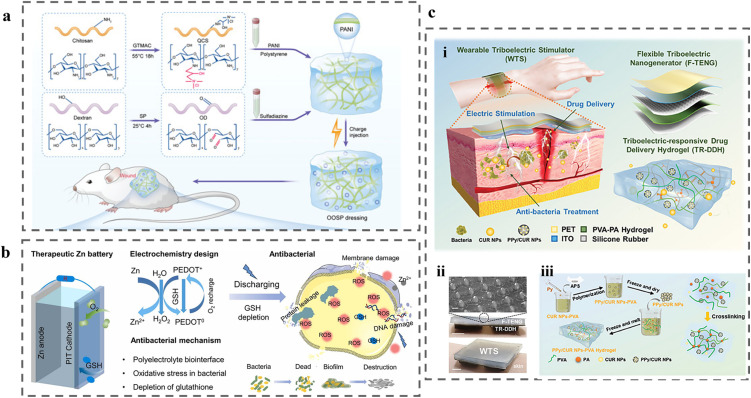
(a) The preparation process of QOSP hydrogel and then the injection of charge into the QOSP hydrogel. Adapted with permission from ref. 269 Copyright © 2025 Wiley. (b) Schematic illustration of tailoring the electrochemistry of the therapeutic Zn battery. During treatment (battery discharge), oxidised PEDOT^+^ is electrochemically reduced to PEDOT^0^ at the Zn anode, accompanied by the generation of H_2_O_2_. Upon exposure to air, the system undergoes an oxygen-driven recharging process. When the battery is in an open-circuit state, the oxidised PEDOT^+^ can deplete intracellular GSH, thereby maintaining elevated ROS levels within bacterial cells and facilitating biofilm eradication. Adapted with permission from ref. 270 Copyright © 2024 Elsevier. (c) Design of the WTS for bacterially infected wound healing. (i) Schematic diagram of the WTS that consists of the F-TENG and the TR-DDH. (ii) Photos of the microstructure on the surface of the silicone rubber film and the structure of the WTS. (iii) Fabrication process of the TR-DDH. Adapted with permission from ref. 271 Copyright © 2024 Wiley.

A chitosan (QCS)/oxidised dextran (OD)/sulfadiazine (SDI)/PANI/polystyrene (PS)/plasma hydrogel, termed QOSP, was reported as a multifunctional dressing designed for burn wounds' healing (Fig. 10a).^269^ The hydrogel was synthesised *via* a Schiff base reaction between QCS and OD, subsequently crosslinked with SDI. It incorporated PANI nanowires and PS to improve electrical conductivity, mechanical stability, and antibacterial properties. An essential advancement in the QOSP hydrogel is the application of high-voltage plasma treatment to inject charges within the polymer matrix. This process promotes the formation of a more homogeneous conductive network, thereby enhancing charge retention, surface potential and dielectric breakdown strength. This electroactive matrix facilitates prolonged charge storage and emulates the natural electric fields of skin, allowing for uninterrupted bioelectric stimulation without external power sources, crucial for enhancing fibroblast proliferation, tissue regeneration and epithelialization. The hydrogel demonstrated superior cytocompatibility, little haemolysis and significant antibacterial efficacy against *Staphylococcus aureus* and *Pseudomonas aeruginosa*, with SDI release enhanced in acidic environments characteristic of infected wounds. In a mouse model of second-degree burn wounds infected with mixed bacterial strains, QOSP markedly surpassed uncharged and non-conductive variations, expediting wound healing, diminishing infection and lowering scarring.

Li *et al.*^270^ developed a wearable patch that functioned simultaneously as a stimulation electrode and a physiological signal recorder for infected and diabetic wound healing. The system was comprised of a zinc (Zn) anode coupled with a PEDOT-based polyelectrolyte hydrogel cathode, enabling self-powered ES without the need for external circuitry (Fig. 10b). The ion–electron dual-conductive hydrogel cathode was fabricated *via in situ* polymerisation of a poly(acrylamide-imidazolium salt) network integrated with PEDOT (termed as ‘PIT’). PIT hydrogel exhibited strong tissue adhesion (56 kPa against porcine skin), high electrical conductivity (33.2 S m^−1^) and low electrode–tissue interfacial impedance (1.04 kΩ at 1 Hz). Under the discharge process (0.38 V), the Zn-PIT patch generated physiologically relevant microcurrents, Zn^2+^ ions and H_2_O_2_, while simultaneously depleting glutathione, collectively inducing severe oxidative stress, membrane disruption and biofilm deconstruction of both *E. coli* and *S. aureus*. The generated endogenous-like electric fields with the current densities in the range of 5–100 µA cm^−2^ promoted fibroblast migration, angiogenesis and collagen deposition while suppressing inflammatory responses, thereby accelerating wound closure. However, the Zn-PEDOT battery still exhibits limited energy retention and cycling stability, as evidenced by the retention of only 52% of its initial capacity after ten discharge–charge cycles. Another study reported a wound patch consisted of a Mg battery with a dual-network MXene (Ti_3_C_2_)-based hydrogel cathode, a bioresorbable Mg anode and a polyvinyl alcohol gel electrolyte.^273^ Mg provides a more negative potential to Zn, resulting in an enlarged output voltage for ES (0.56–0.68 V) and was capable of retaining ∼85% of its initial capacity after 1000 stimulation cycles.

Triboelectric nanogenerators (TENGs) have significant potential as autonomous devices for administering therapeutic ES to facilitate various phases of wound healing. Qin *et al.*^271^ reported a wearable triboelectric stimulator (WTS) that consisted of a flexible TENG (F-TENG) and a triboelectric-responsive drug delivery hydrogel (TR-DDH) for the healing of bacterium-infected wounds (Fig. 10c). The working mechanism of the F-TENG arises from the coupled effects of triboelectrification and electrostatic induction. Initially, the silicone rubber contacted the indium tin oxide (ITO), which resulted in charge transfer from the ITO to the silicone rubber, and accordingly, the silicone rubber was negatively charged. When the PET–ITO film begun to move away, the surface of the PVA–PA hydrogel continuously generated a positive charge due to electrostatic induction, which created a potential difference between the ITO and the PVA–PA hydrogel, resulting in a current in the electric circuit. The subsequent contact process between the ITO and the PVA–PA hydrogel generated a reverse current with an alternating current output during periodic contact-separation movements. Pulsed electrical stimulation *via* the F-TENG enabled the controllable release of curcumin (CUR) NPs from the PPy. The result also showed that the release rate and efficiency of the CUR NPs in the WTS were approximately twofold higher than those of the group not subjected to ES. The results of *in vitro* and *in vivo* experiments revealed that the current range of 2–4 µA produced by WTS significantly promoted cell migration, inhibited the expression of the proinflammatory cytokines nterleukin-1 (IL-1), tumour necrosis factor-α (TNF-α) and interleukin-6 (IL-6), and induced the expression of the anti-inflammatory interleukin-10 (IL-10).

Recently, CPs have been integrated with biofuel cells (BFCs), where CPs are primarily used as polymeric matrices for enzyme immobilization.^274^ BFCs generate green electricity from energy-dense carbon-neutral fuels like glucose or lactate in wound fluid.^274^ At present, CP-BFCs integration has predominantly been explored in modular configurations, where CPs function as separate sensing or drug-delivery elements. For example, PANI polymers showed readable colour change when coupled with a glucose oxidizing bioanode for glucose sensing.^275^ PEDOT:PSS was also recently used as an external chromic display for glucose and lactate sensing.^276^ In parallel, CPs have also been demonstrated as electro-responsive drug reservoirs in BFC-powered systems.^277^ For instance, the release of the drug (anionic acetaminophen) based on CP-BFCs has been achieved using a lactate-oxidizing bioanode and a PEDOT-based cathode loaded with the drug.^278^ In another study, a controllable release of both anionic and cationic model compounds was demonstrated on an Os redox polymer mediated CP-BFCs by appending an additional PEDOT or PPy/drug layer onto a O_2_ reducing biocathode.^279^

Closed-loop wound dressings enable precision personalised medicine.^280^ Closed-loop wound dressings typically contain four fundamental components: (1) sensors for collecting wound condition parameters; (2) algorithms for analysing input signals and issuing desired intervention commands; (3) controllable therapeutics systems and (4) wireless communication modules for data transmission.^281^ As discussed above, CP-based wound dressings inherently support multifunctional therapeutic modalities and can be readily integrated with self-powered architectures, positioning them as promising platforms for closed-loop wound management. Beyond local feedback, advances in low-power computation and wireless communication have enabled distributed closed-loop regulation and user interfacing in emerging electroceutical wound dressings.^282,283^ These developments provide an enabling framework for CP-based systems, which are discussed below.

**Fig. 11:**
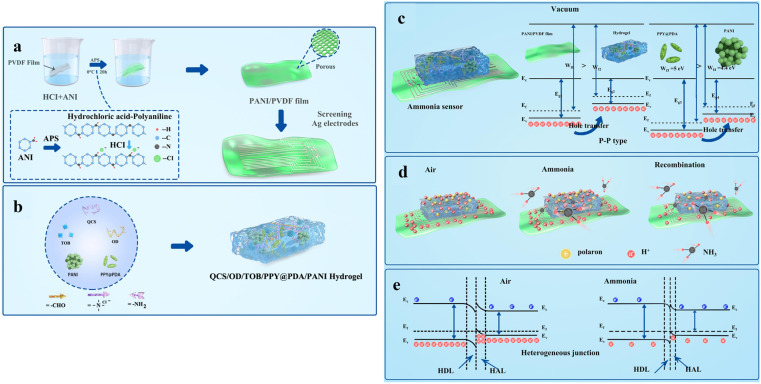
The multifunctional dressing PPy@PDA/PANI/PVDF design (a and b) and gas sensing mechanism (c–e). Preparation of (a) PANI/PVDF film and (b) PPy@PDA/PANI hydrogels; (c) The formation of a p–p heterojunction interface between PPy@PDA and PANI, a hole accumulation layer (HAL) forms on the surface of the sensitive material. Meanwhile, according to the formation of the hole depletion layer at the p–p heterojunction interface, the charge concentration (conductivity) HAL at the surface (shell layer) is higher than that in the inner (core) portion, which leads to the migration of charge carriers at the semiconductor surface. (d) When the PPy@PDA/PANI hydrogel sensor is exposed to ammonia gas, the gas is adsorbed onto the surface of the material. (e) The electrons released by the ammonia gas interact well with the holes in the valence band of the sensitive material, narrowing the thickness of the hole accumulation layer and resulting in an increase in the sensor resistance, which reflects the sensing response. Adapted with permission from ref. 153 Copyright © 2023 Elsevier.

A PPy@PDA/PANI hydrogel integrated with PANI/polyvinylidene fluoride (PVDF) film was developed for real-time ammonia sensing and an electrically regulated drug for infected wound management (Fig. 11).^153^ This system comprised a real-time wound monitoring module, an electrical stimulation parameter module, a drug release module and a communication module. This system demonstrated a highly porous architecture with evenly dispersed PPy@PDA NWs, offering a substantial specific surface area (∼64 µm pore size) and multiple active sites favourable for effective ammonia sensing. The best configuration, PPy@PDA/PANI (3/6), exhibited superior ammonia-sensing capability, characterised by a rapid reaction time of 23.2 s, a swift recovery time of 42.9 s and a sensitivity of 23.5% at 1 ppm NH_3_, with a theoretical detection limit of 49 ppt. Remarkable selectivity against other gases, reliable performance under diverse humidity and pH conditions and a steady charge-transfer-based sensing mechanism were reported. A wireless app-controlled system enabled real-time monitoring of ammonia concentration and precise regulation of electrically triggered medication release, therefore establishing a closed-loop feedback system to assure appropriate dose.

Another study demonstrated integrated closed-loop continuous impedance and temperature monitoring with responsive ES to the evolving wound environment.^272^ The system was built on a flexible printed circuit board (FPCB) incorporating an energy-harvesting antenna, a microcontroller unit, a crystal oscillator and filter circuits for dual-channel continuous sensing of wound impedance and temperature, a parallel stimulation circuit to deliver programmed electrical cues for accelerated wound healing, as well as a tissue-interfacing PEDOT:PSS-based conducting adhesive hydrogel interface for robust and gentle skin integration for effective ES. The hydrogel exhibited strong skin adhesion at physiological temperature, but its interfacial adhesion decreased reversibly by approximately two orders of magnitude when heated to 40 °C. The smart bandage operated wirelessly *via* inductive coupling to an external radiofrequency identification (RFID) reader, which powered both electrical stimulation and near-field communication (NFC)-based data transmission. The smart bandage was able to monitor temperature and impedance changes at the wound site continuously. Across mouse wound models, this closed-loop system achieved ∼25% faster wound closure and ∼50% enhancement in dermal remodelling relative to controls.

The above studies demonstrate that CP-based wound dressings have great potential to integrate on-demand treatment, continuous monitoring and adaptive wound management within unified platforms, pawing a way toward a new generation of closed-loop, responsive and personalized wound care systems.

## 7. Conclusions and outlook

In summary, the evolution of CP-based wound dressings from single-function electroactive materials to multifunctional, self-powered and closed-loop platforms reflects a paradigm shift toward intelligent wound management. The combination of ES and drug delivery in CP-based wound dressings synergistically enhances the healing outcome, while further integration of CP-based sensors allows real-time monitoring of wound biomarkers and the precise regulation of drug release and/or ES. Continued progress in CP materials and device engineering will facilitate the integration of self-powered operation, wireless communication and intelligent data analytics, enabling autonomous, adaptive CP-based systems capable of real-time modulation and optimisation of wound healing.

Despite these advances, a number of challenges remain before CP-based multifunctional wound dressings can be translated into practical, large-scale applications. These can be broadly categorised into four key aspects: (i) conductivity and long-term electrochemical stability: progressive over-oxidation, dopant loss and microstructural degradation can compromise conductivity, charge-injection capacity and sensing fidelity during prolonged operation. (ii) Biodegradability: the intrinsic chemical stability of π-conjugated backbones restricts CP degradation under physiological conditions, limiting their suitability for wound healing applications. (iii) Multifunctional system integration: the incorporation of sensing, stimulation, drug delivery and energy modules within a single compact platform increases structural complexity, interfacial instability, power-management challenges and risks of signal interference between components. (iv) Clinical translation and regulatory considerations: issues including large-scale reproducible manufacturing, sterilization compatibility, long-term biosafety evaluation and compliance with medical-device regulatory frameworks remain significant barriers to commercialization.

Based on the above challenges, the development of CP-based wound dressings should also incorporate the following considerations and could align with the following strategies.

Chemical safety represents a critical yet often underexplored consideration that is intrinsically coupled to electrochemical stability. Degradation processes such as overoxidation, dopant leaching and structural breakdown not only impair material performance but may also introduce chemical risks within the wound microenvironment. These include cytotoxic effects arising from residual monomers and oxidants (*e.g.*, pyrrole, aniline, Fe^3+^ or persulfate systems), disruption of local ionic balance due to the leaching of small-molecule dopants (*e.g.*, Cl^−^, *p*-toluenesulfonate) and the formation of reactive or acidic degradation byproducts that may alter pH, induce oxidative stress or trigger inflammatory responses.^284,285^ To mitigate these risks, some rational chemical design strategies have been developed. The use of macromolecular dopants, such as PSS, hyaluronic acid or heparin, enhances electrostatic retention and reduces dopant diffusion while improving hydrophilicity and biocompatibility.^12,13^ For instance, HA doped PPy electrodes, showed higher hydrophilicity leading to better wetting, the abundant negative charges aiding the formation of more ordered PPy structures during deposition, and the prevention of de-doping in the medium.^13^ Crosslinking and network stabilisation, *via* chemical bonds or supramolecular interactions, further suppress the release of low-molecular-weight species and enhance structural robustness under repeated redox cycling.^182^ Moreover, physically adsorbed biomolecule drugs within CPs are often pH-sensitive and may leach into the surrounding medium, compromising both therapeutic control and electrodes' stability.^286^ Covalent conjugation of drug molecules directly onto CP-based dressings could enhance the precise control over therapeutic release. Unlike physically entrapped or ionically doped therapeutics, covalently bonded drugs have a more stable connection to the material, thereby preventing uncontrolled drugs leaching and stabilizing the electroactive surface. Post-synthetic purification and conditioning processes, including solvent washing, dialysis and electrochemical pre-treatment, are also essential for removing residual monomers, oxidants and loosely bound dopants.^72^

CP-based dressings must retain mechanical durability under continuous deformation, hydration–dehydration cycles, and redox-induced volumetric changes.^26^ Accordingly, future design strategies could focus on reinforced polymer composites, dynamic or reversible crosslinking chemistries and hybrid composite architectures that decouple electrochemical functionality from mechanical integrity, thereby enabling self-healing, long-term durability and structural stability without sacrificing electrochemical performance.

The development of degradable CP systems has emerged as an important direction for improving long-term biocompatibility by enabling controlled breakdown and reducing long-term material persistence. Although CPs such as PPy, PANI and PEDOT exhibit excellent electrochemical performance, their non-degradable conjugated backbones raise concerns regarding material persistence in biological environments. Recent reviews discuss concepts of CP-based transient electronics for biomedical applications,^287,288^ where CPs are expected to be engineered to retain electroactivity during the critical wound-healing period and subsequently degrade into biologically benign fragments, thereby minimising long-term material persistence. These reviews discuss strategies towards degradability,^287,288^ including the incorporation of cleavable linkages (*e.g.*, ester, imine/Schiff base, acetal/ketal and disulfide bonds) either within the polymer backbone or as pendant functional groups. These motifs enable hydrolytic, enzymatic or redox-responsive degradation under physiological conditions. Additionally, as discussed earlier, Beikzadeh *et al.*^114^ reported a disulfide-based chemistries in CP systems to enable electrochemically controlled cleavage for drug release, which also potentially provide a promising strategy for on-demand degradation. Copolymerisation and composite approaches, in which conjugated segments are combined with biodegradable non-conjugated domains, provide means to retain partial electroactivity while enabling structural disintegration. For example, porous PPy/chitosan scaffolds exhibited significant enzymatic degradability,^289^ with 35–40% weight loss *in vitro* over 10 days and corresponding conductivity reduction (10^−2^ to 10^−6^ S cm^−1^), while *in vivo* studies showed gradual mass loss (to 68–80%) and decreased conductivity (10^−2^ to 10^−4^ S cm^−1^). It was also suggested that 3–6 wt% of PPy in the scaffold should be suitable for practical tissue engineering applications. However, a fundamental trade-off persists between maintaining extended π-conjugation for efficient charge transport and introducing chemically labile bonds for degradation.^290^ As a result, most current systems rely on degradable non-conjugated components, while fully degradable conjugated backbones capable of yielding non-toxic, well-defined metabolites remain an unresolved challenge. For example, in PPy/PDA/poly(l-lactide) (PLLA) membranes,^291^ the PLLA component undergoes hydrolysis that generates lactic acid, which can lower local pH and potentially trigger inflammatory responses if not adequately managed. Therefore, future research must not only focus on balancing the biodegradability and conductivity, but also on tuning degradation behaviours and ensuring that byproducts do not adversely affect wound healing outcomes.

To advance the efficacy and clinical potential of CP-based wound dressings, next-generation design strategies should focus on responsive functionality and regenerative microenvironment control. One novel approach is the incorporation of multi-modal, spatiotemporally controllable drug delivery systems into the conductive scaffold.^292,293^ For example, PDA-doped CP/nanozyme hydrogels,^261,262^ offer dual responsiveness, combining ROS- or pH-sensitive release with ES-triggered release. In these systems, the hydrogel matrix undergoes structural or swelling changes in response to wound-associated pH and ROS fluctuations, while the incorporated CPs enables electroresponsive release of embedded nanozymes. This design supports both autonomous, condition-adaptive release and externally programmed, spatially localised therapeutic modulation. Nevertheless, further refinement toward fully programmable platforms remains necessary, such as (i) coupling with mild ES to trigger release of therapeutics that can be dynamically adjusted according to wound stage or biomarker feedback; (ii) in combination with photodynamic therapy (PDT), sonodynamic therapy (SDT) and photothermal therapy (PTT) could potentiate the effects on the ES efficiency and contribute to enhanced biofilm inhibition;^294,295^ (iii) using gradient conductivity designs, where conductivity increases toward the wound core to promote directed cell migration and tissue regeneration, would be another advanced approach to increase efficacy of wound healing dressings.^296^

Electrospinning, 3D printing and advanced manufacturing technologies enable anisotropic architectures (aligned nanofibers^297^ or patterned CPs^298^) that can also enhance electro-guided cellular behaviour, especially for re-epithelialization and angiogenesis. These architectural and release features could be integrated with real-time biosensing elements (for pH, temperature, metabolites or cytokines) that can inform both therapy and clinical decision-making. As introduced above in Section 7, a hierarchically porous PPy@PDA/PANI hydrogel integrated with a PANI/PVDF film^153^ was engineered to enable real-time ammonia sensing coupled with electrically regulated drug release, which demonstrated the application on integrating sensing and electro-responsive therapy for feedback-regulated infected wound management.

Although self-powered designs of CP-based wound dressings are attractive for autonomous operation, they present challenges in energy conversion and storage efficiency. A promising strategy involves integrating photothermal materials into PEDOT-based hydrogels, enabling near-infrared (NIR) light to be converted into localised heat within the electroactive matrix.^299^ The elevated temperature enhanced charge carrier mobility in the PEDOT backbone and accelerated ionic transport in the hydrated network, leading to increased electrical conductivity and reduced internal resistance. In addition, thermally induced temperature gradients can contribute supplementary voltage *via* the thermoelectric (Seebeck) effect, leading to more stable and efficient, sustainable, low-voltage ES.^300^

Many CP-based dressings demonstrate short-term cytocompatibility and antibacterial activity. However, the consequences of their prolonged presence are rarely studied.^301^ To date, most *in vivo* studies of CPs have been conducted in animal models, with only a limited number involving human subjects. However, studies on CPs in clinical settings have shown promising progress. For instance, PEDOT:PSS microelectrode arrays have been used to record the neural activity in 37 human participants, demonstrating high sensitivity in detecting localised cortical events that conventional clinical electrodes failed to capture.^302^ Amplicoat®, a PEDOT-based coating designed to improve the performance of metal and polymer substrates particularly in biomedical and high-performance applications, has recently received approval from the US Food and Drug Administration (FDA) and the Conformité Européenne (CE) mark.^303^ The success of Amplicoat® will serve as a valuable model for the translation of CPs into clinical applications. In parallel, alternative conductive materials, such as carbon–metal and metal oxide-based materials,^304,305^ are also being actively explored in clinical applications due to their established manufacturing pipelines and regulatory familiarity. Realistically, multifunctional CP-based wound dressings are likely to require longer development timelines, due to the need for reproducible synthesis, sterilisation compatibility, long-term stability data and regulatory validation.^12^

Looking forward, CP-based wound dressings are gradually transitioning toward fully integrated intelligent systems. Emerging studies on CP-based sensors that couple local wound sensing with systemic electrophysiological monitoring modalities, such as electrocardiography (ECG), electromyography (EMG) and electroencephalography (EEG),^306^ provide a pathway toward comprehensive, system-level feedback on physiological states relevant to wound healing. Artificial intelligence (AI) is expected to play a transformative role, enabling anomaly detection, predictive modelling and personalised therapy guidance.^281,307^ For example, Ward *et al.* implemented AI in bioelectronics to mitigate antibiotic side effects.^308^ They developed a closed-loop wound patch integrated with a pyocyanin sensor, an artificial neural network (ANN)-assisted antibiotics toxicity prediction and controllable dosing modules. Kalasin *et al.* reported that pH-responsive electrical signals from a conductive hydrogel dressing can distinguish wound healing stages, with characteristic potentials (985, 496 and 48 mV) corresponding to inflammation, proliferation and remodelling, respectively.^309^ Coupled with a deep learning ANN model, the system achieved high accuracy (∼94.6%) in classifying wound states and predicting healing progression. Wang *et al.* developed a microfluidic wearable device for real-time wound exudate analysis, capable of detecting pH, temperature, and reactive species (*e.g.*, O_2_, NO, H_2_O_2_) within 1 min. The device integrates a pH sensor based on an electropolymerised PANI film as the sensing component and achieves high accuracy (88.75–94.0%) in quantifying wound biomarkers. Furthermore, the device could determine the correlation between biomarker concentrations, wound status and patient health conditions with the aid of well-trained AI models (*e.g.*, K-nearest neighbours, radial basis and support vector classification).^310^ Consequently, these advances highlight the growing potential of AI-assisted, closed-loop platforms to deliver continuous monitoring, predictive diagnostics and personalised therapeutic interventions, thereby advancing the development of intelligent and adaptive wound-care systems. Concurrently, advances in four-dimensional (4D) bioprinting have enabled the fabrication of a variety of stimuli-responsive materials, such as electrical, thermal, humidity, pressure and photo-responsive materials, to create smart dressings that can transform structurally and respond to internal and external stimuli for post-printing functionality, as well as possess the environmental and structural dynamics of native tissues.^311,312^ Compared to 3D printed components that remain relatively static, 4D printed structures can transform into another shape or configuration when subjected to external stimuli. Consequently, the 4D printed structures possessed enhanced structural and biological functionality.^312^ For example, Wang *et al.* reported a 4D-printed MXene-based shape-memory nerve conduit that autonomously rolls at physiological temperature (37 °C) to wrap nerve stumps, while its conductive microchannel architecture facilitates cell migration and electrical integration, achieving functional recovery comparable to autografts.^313^ Beyond implantable systems, 4D bioprinting has also been integrated with *in vitro* wound-healing models, particularly through the co-engineering of hydrogel-based drug delivery platforms with microfluidic and biological components. These platforms can recapitulate the complex biochemical and mechanical microenvironment of human tissues, enabling more physiologically relevant evaluation of therapeutic responses.^314^ For instance, 4D-printed hydrogels incorporated into skin-on-chip systems have been designed to release antimicrobial agents in response to pH variations associated with infection, allowing real-time monitoring and intervention. Similarly, shape-morphing hydrogel constructs have demonstrated spatially controlled delivery of growth factors, aligning therapeutic release with the sequential stages of wound healing.^315^ Although these systems are not based on CPs at present, they provide relevant design principles for stimuli-responsive and adaptive wound healing platforms, which are conceptually aligned with CP-enabled functionalities.

In summary, continued convergence of medical materials science, bioelectronics, data science, AI and advanced manufacturing is expected to drive the emergence of intelligent, adaptive and patient-specific wound-care platforms.

## Author contributions

Jingwen Yang prepared the initial draft of the manuscript and conducted the primary literature analysis. Lisa I. Pilkington and Jadranka Travas-Sejdic contributed to the conceptual development of the review, provided guidance and supervision throughout, and were actively involved in revising and refining the manuscript. All authors contributed to the final editing and approved the submitted version.

## Conflicts of interest

The authors declare no conflicts of interest.

## Data Availability

No primary research results, software or code have been included, and no new data were generated or analysed as part of this review.
